# A cascade targeting strategy based on modified bacterial vesicles for enhancing cancer immunotherapy

**DOI:** 10.1186/s12951-021-01193-9

**Published:** 2021-12-20

**Authors:** Yuewen Zhai, Yuying Ma, Bo Pang, Jinnan Zhang, Ying Li, Yalan Rui, Tian Xu, Yu Zhao, Zhiyu Qian, Yueqing Gu, Siwen Li

**Affiliations:** 1 State Key Laboratory of Natural Medicines, Jiangsu Key Laboratory of Drug Screening, Department of Biomedical Engineering, School of Engineering, China Pharmaceutical University, No. 639 Longmian Avenue, Jiangning District, Nanjing, 211198 China; 2 Department of Neurosurgery, China-Japan Union Hospital, Jilin University, Changchun, Jilin China; 3 Department of Biomedical Engineering, School of Automation, Nanjing University of Aeronautics and Astronautics, 29th JiangJun Street, Nanjing, 211106 Jiangsu China; 4 Jilin Ginseng Academy, Changchun University of Chinese Medicine, Changchun, 130117 China

## Abstract

**Background:**

As an efficient tumor immunotherapy, PD-1 antibody has been gradually used in clinical tumor treatment, but the low response rate and excessive immune response limit its extensive application.

**Results:**

Herein, a therapeutic regime for the reinvigoration and activation of the tumor immune microenvironment is introduced to improve the anti-tumor effect of the PD-1 antibody. To comprehensively improve the effect of the immunotherapy and reduce excessive immune response, a biomimetic cascade targeting nanosystem, siRNA@PLOV, which was fused by photothermal sensitive liposomes (PTSLs) and attenuated *Salmonella* outer membrane vesicles (OMVs), was administered in the tumor therapy for targeting of tumor tissues and T cells within tumor respectively. The fused PLOVs which not only retained the biological character of the OMVs, but also enhanced the drug loading ability. The results demonstrated that the immunogenicity of OMVs and photothermal effects can obviously increase the infiltration of T cells and the silencing of CD38 can effectively improve the T cell cytotoxicity, especially combining with PD-1 antibody.

**Conclusions:**

Interesting, this study revealed that anti-PD-1 administration on the 5th day after siRNA@PLOV treatment had the best performance in killing tumors compared with other groups. In addition, this new therapeutic regime also presents a novel strategy for inducing “vaccine effects”, conclusively highlighting its potential in preventing tumor recurrence and improving prognosis.

**Graphical Abstract:**

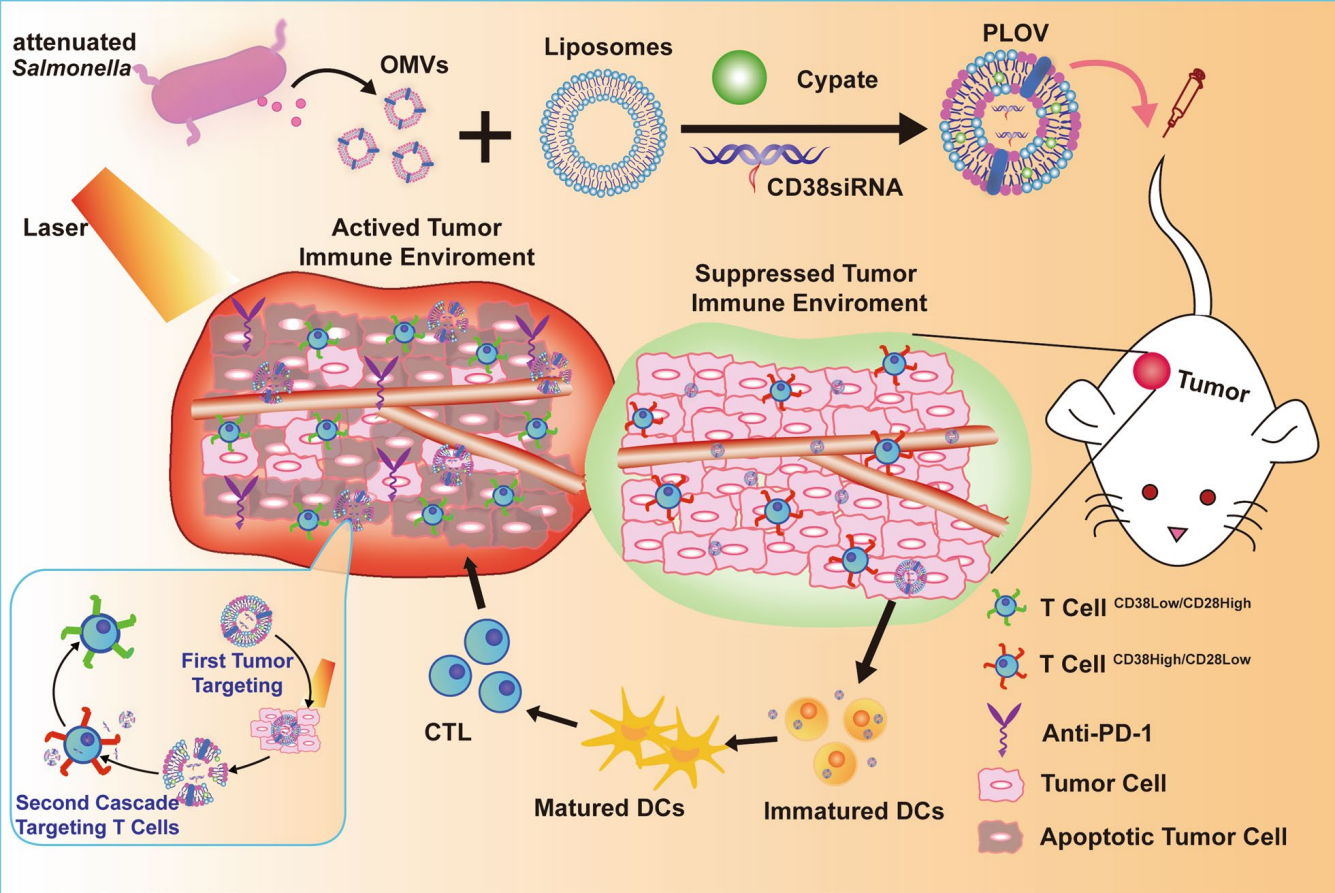

**Supplementary Information:**

The online version contains supplementary material available at 10.1186/s12951-021-01193-9.

## Background

Cancer immunotherapy has emerged as a next-generation strategy to attenuate tumor cells by regulating the tumor immune microenvironment [1–4]. Aimed at blockading immune checkpoint molecules to inhibit tumor evasion, immune checkpoint therapy, a new cancer immunotherapy paradigm, has successfully given rise to an endurable immune response in many types of tumors [5]. Programmed death-1 (PD-1) antibody, an immune checkpoint inhibitor, has exhibited remarkable curative effects, owing its great success to the utilization of anti-PD-1 axis as a first-line therapeutic in immunotherapy [6–9]. However, the clinical benefits of the PD-1/PD-L1 antibody are only available to a minority of the population [10].

In recent studies, low treatment response rate and low tumor mutation burden have been shown to have a close relationship [11]. Due to low tumor mutation burden, there is increased difficulty in the recognition of tumor cells by corresponding immune cells [12]. Moreover, the immunotherapeutic effect of PD-1 antibody may be restricted by the tumor immune microenvironment [13, 14]. In the early stages of tumorigenesis, CD8+ T cells are effectively activated by the PD-1 antibody, however, in the late-stage tumor immune microenvironment, dysfunction of tumor-specific CD8+ T cells may inevitably become irreversible [15, 16]. CD38, a major mammalian NAD+ glycohydrolase (NADase), expresses on T cells following activation. The importance of CD38 in regulating T cell function is that deplete intracellular NAD^+^ level and generates key signaling mediator, cADPR in T cells concomitantly [17]. The cADPR catalyzed by CD38 can be further transformed into adenosine, a well-known small molecule metabolite that plays an immunosuppressive function, under the action of CD203, CD73 and other enzymes. During the early stages of tumorigenesis, it is also reported that CD8+ T cells express low level of CD38, while expression levels remain extremely high during the advanced stages [18, 19]. Based on the significant difference of CD38 expression between early and late stages of tumorigenesis, it was hypothesized that CD38 expression was consistent with the therapeutic efficacy of PD-1 antibody [20]. Therefore, in the present study, the strategy of down-regulating CD38 expression was developed in combination with PD-1/PD-L1 blocking antibodies to enhance the treatment effects of immunotherapy. Herein, we found that reduced expression of CD38 can effectively improve the therapeutic effects of the PD-1 antibody.

Photothermal therapy (PTT), a tumor treatment modality, utilizes the heat energy generated from photothermal conversion by photosensitive reagents [21–23]. PTT allows for easier recognition of T cells by enhancing antigen exposure of tumor cells via a systemic anti-tumor immune response including the translocation of calreticulin (CRT) from the endoplasmic reticulum to the cell surface, the release of high mobility group B1 from the nucleus, the extracellular secretion of adenosine triphosphate and the expression of heat shock proteins (HSP) on the cell surface [24–26]. In the recent years, bacterial outer membrane vesicles (OMVs), prokaryotic vesicles shed from Gram-negative bacteria, have been applied in biotherapeutics or biomimetic carriers. OMVs contain many natural adjuvant components inherited from the parent bacterium but in a non-replicative form, which can stimulate immune maturation and initiate inflammation in a controllable manner. The consequent inflammatory environment triggered by PTT and OMVs increases immune cell infiltration, serving as an artificial replication of the initial tumor immune microenvironment thereby establishing early-stage immune circumstances that are beneficial for immunotherapy [27, 28].

However, as excessive immune response can induce unnecessary side effects such as high fever induced by immunocytokines in immunotherapy, it is of great significance to reduce the occurrence of excessive systemic immunity [29–32]. To approach the issue of excessive immune response, a cascaded double-target biocamouflage liposome-based nanosystem was constructed with the ability to carry the loaded cargos to the tumor tissues and targeting the T cells respectively. A single-chain antibody of CD7 conjugated with a nona-d-arginine (9R) peptide complex was then used to facilitate the targeted delivery of CD38 siRNA into T cells [33–36]. Once exposed to near-infrared light in tumor tissues, the anti-CD7-9R complexes within the fusion biomimetic carriers are released in response to structural decomposition and uptake by T cells due to receptor-mediated endocytosis based on CD7 and cell piercing peptide 9R. Hence, the drug delivery system could target the tumor tissues and the T cells within the tumor site by a cascaded way.

In this study, as shown in Scheme, biomimetic nanocarriers (PLOVs) are constructed by fusing of OMVs and PTSLs. In vivo, due to OMVs self-adjuvanting and thermal effect produced by PTSLs under NIR irradiation, PLOVs promoted immune maturation and improved tumor immune environment. Concurrently, CD38siRNA inhibition can enhance the effect of T cells in tumor environments. By detecting tumor immune environments at different stages, PD-1, an immune checkpoint inhibitor, was combined with better immune environment on the 5th day after PTT to achieve excellent efficacy in the treatment of primary or distal tumor and metastasis. Therefore, our treatment strategy in this study will provide new ideas and basis for clinical tumor hyperthermia combined with immunotherapy.

## Results and discussion

### Synthesis and characterization of siRNA@PLOV

**Fig. 1:**
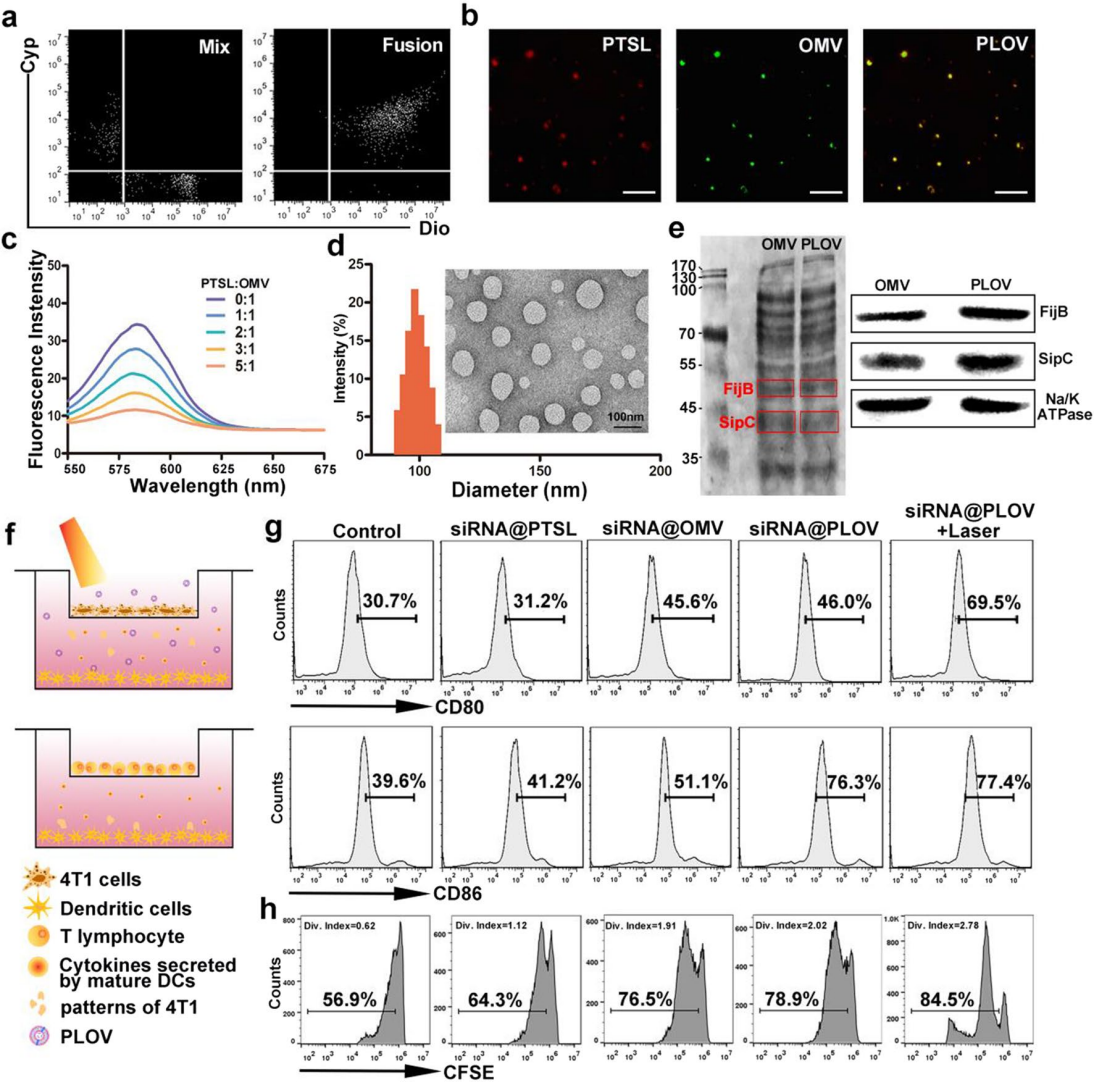
Synthesis and characterization of siRNA@PLOV. **a** Flow cytometry analysis of Cyp versus Dio intensity in the mixture or fused of OMVs and PTSLs. **b** CLSM images of PTSL (Cyp), OMV (Dio) and PLOV. **c** A pair of Förster resonance energy transfer (FRET) fluorescence dye DiO and DiI is chosen to conduct the fusion process of OMVs with the increasing amounts of PTSLs. **d** Hydrodynamic size and TEM images of the resulting PLOV. **e** SDS-PAGE and western blot protein analysis of FijB and SipC from OMVs and PLOV. **f** Schematic illustration of the transwell system experiment. The 4T1 cells are cultured in the upper chamber and BMDCs are cultured in the lower chamber. After 4T1 cells with treatments of laser, BMDCs are collected for analysis or transported to another transwell system with T cells in upper chamber. **g** The percentage of DCs derived of the flow cytometry analysis of CD80 and CD86. **h** CFSE-labeled T cells (10^5^) are incubated with BMDCs which has been treated with formulations (10^3^ particles/mL), and the proliferation of T cells is analyzed by flow cytometry

In order to achieve the purpose of cascade targeting and successfully delivering more CD38 siRNA to the tumor tissues, a fusion biomimetic nanocarrier was constructed by fusing OMVs and photothermal sensitive liposomes (PTSL) based on the similarity-intervisibility theory, which not only retained the biological characteristics of the OMVs, but also enhanced the ability of drug loading. The PLOVs are engineered by fusing OMVs of attenuated *Salmonella* and photothermal sensitive liposomes (PTSLs). Cypate, a hydrophobic fluorescent dye, was incorporated into bilayer thermosensitive liposomes to generate heat (Additional file 1: Fig. S1). The OMVs were stained with Dio, to verify the fusion of OMVs and PTSLs. As shown in Fig. 1a, the PLOVs exhibited the double positive fluorescent intensity. As shown in the confocal laser scanning microscopy (CLSM) images, the PLOVs exhibited significant colocalization of fluorescence signals when fusing the OMVs and PTSLs (Fig. 1b). In Fig. 1c, the OMVs were labeled with a pair of dye (DiO/DiI: Ex484; Em501/Ex549; Em565) and DiI signal (565 nm) could be detected when irradiated with 484 nm laser as the FRET effect. With increasing amounts of PTSLs fused, the fluorescence intensity of DiI decayed as the distance of DiO/DiI was increased, suggesting that the PTSLs intrude into OMVs successfully. The hydrate particle size, transmission electron microscopy and Zeta potential of nanoparticles were detected including PLOVs, OMVs and PTSLs (Fig. 1d and Additional file 1: Fig. S2). SDS-PAGE and western blot protein analysis showed that PLOVs retained the characteristic protein of OMVs (Fig. 1e). Anti-CD7-9R complexes were uploaded to PLOVs to achieve more effective treatment. The amount of anti-CD7-9R loaded in PLOVs was increased compared to OMVs (Additional file 1: Fig. S3a). And then the siRNA@PLOV also showed the good stability and drug release rate in different circumstance (Additional file 1: Fig. S3b–d). To investigate self-adjuvating effects of OMVs and PTT, a transwell system was designed (Fig. 1f). Particularly, the proportion of mature BMDCs in the siRNA@OMV and siRNA@PLOV groups is higher than that in the siRNA@PTSL group, indicating the potent adjuvating activity of OMVs ingredient, while upon the photothermic induced 4T1 cell death, the activated BMDCs are further increased in the siRNA@PLOV groups compared with the treatment groups without irradiation (Fig. 1g, Additional file 1: Fig. S4). siRNA@PLOV with laser treatment also demonstrated increased activated DC cells in the 4T1 solid tumor model in vivo (Additional file 1: Fig. S5). Meanwhile, the mature BMDCs can process tumor antigens and subsequently initiate the antitumor immune response by secreting cytokines such as TNF-α and IL-12. The enzyme-linked immunosorbent assay (ELISA) quantification reveals that siRNA@PLOV with NIR irradiation treatment shows about 3.7 times larger stimulation on cytokine expression compared with siRNA@PTSL treatments, suggesting a superior activation on DC functionalization (Additional file 1: Fig. S6). Since IL-12 is responsible for activating the cytotoxic T lymphocytes (CTLs)-based immunity, the in vitro T cell proliferation by the treated BMDCs is further evaluated via flow cytometry. As shown in Fig. 1h, compared with the untreated control group (6.98%), the siRNA@PLOV and with NIR irradiation treatments induce a significant increase of T cell proliferation to 12.9 and 20.1%. As evaluating the bio-distribution of siRNA@PLOV in vivo, the massive accumulation of siRNA@PLOV was clearly observed at 4 h post-intravenous injection (Additional file 1: Fig. S7a). The real-time thermal images displayed that the temperature in the tumors of the mice treated with PLOV quickly rose to 58 ℃ within 6 min (Additional file 1: Fig. S7b). Next, the cell uptake towards siRNA@PLOV were monitored (Additional file 1: Fig. S8). As revealed in Additional file 1: Fig. S9, the survival rates of 4T1 and L02 cells were monitored, and siRNA@PLOV of 2.0 nM were utilized as the maximum safe concentration in the later research. The in vivo toxicity of the siRNA@PLOV was further testified by H&E staining, revealing negligible toxicity of the carriers to body (Additional file 1: Fig. S10).

### The infiltration capacity of immune cells in the distal tumor sites at different time points post PTT based on PLOV

**Fig. 2:**
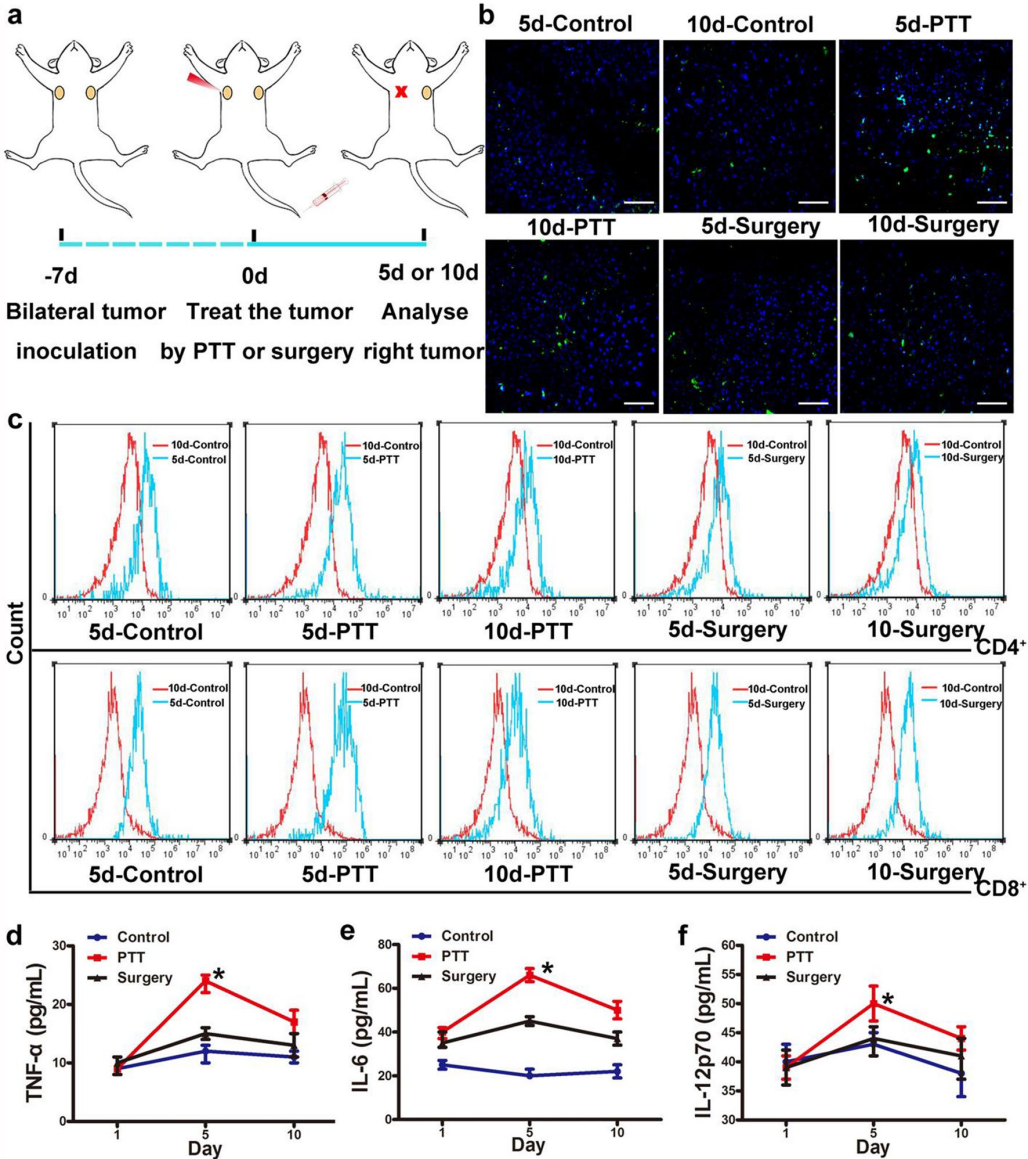
The infiltration capacity of immune cells in distal tumor sites at varied time points after PTT. **a** Schematic illustration of PLOV-based PTT to suppress tumor growth at distal tumor. **b** Immunofluorescence of CD8+ T cells (FITC labeled anti-CD8+) in tumor tissues of different treatment groups. The scale bar is 100 μm. **c** Flow cytometry analysis of CD4+ and CD8+ T cells in the distal tumor tissues under various treatments. **d**–**f** The changes of various cytokines in the distal tumor tissues of mice bearing H22 tumors, including TNF-α (**d**), IL-6 (**e**) and IL-12p70 (**f**) were examined by ELISA at day 1, day 5 and day 10 after respective treatment (PBS, surgical resection and PTT). Data are given as mean ± SD (n = 7). Statistical analysis was conducted by the Student t test for two groups and the one-way ANOVA for multiple groups, and the statistical significance was set as *P < 0.05

Results from primary tumors stimulated with PTT exhibited systemic anti-tumor immune response. While it is noteworthy that systemic anti-tumor activity can be efficiently enhanced by an immune checkpoint inhibitor, the optimum administration time of an immune checkpoint inhibitor post-PTT required further investigation. As illustrated in Fig. 2a, H22 tumor cells were inoculated on the bilateral side of the mice. Inoculation of the H22 tumor cells at the left side served as the site of the primary tumor, while the right functioned as an artificial imitation of tumor metastasis. The primary tumor on this mouse was eliminated by PTT under the NIR irradiation laser.

Mice which received treatment via single surgical resection of the primary tumor served as a control group. To assess the abscopal effect elicited by PTT and surgery on the primary tumor, the levels of CD4+ T cells and CD8+ T cells were measured in the distal tumor site of the mice on the 5th and 10th day post-treatment respectively. Immunofluorescence by green fluorescence signal of CD8+ T cells (FITC-labelled anti-CD8+) in the distal tumor, represented by immunofluorescence assay, demonstrated that compared with the corresponding PBS or surgery groups, PTT treatment groups illustrated the enhanced infiltration of CD8+ T cells. The degree of lymphocyte infiltration was extensive on the 5th day and declined on the 10th day after PTT treatment (Fig. 2b). Substantial reduction of infiltrating CD8+ T cells into distal tumor was also observed on the 10th day after PTT as compared to the 5th day. Density of CD8+ T cells in tumors in different groups keep consistence with the above results (Additional file 1: Fig. S11).

Immunofluorescence assay revealed that the infiltration of CD8+ T cells were dramatically enhanced in the distal tumor of mice post-PTT on the 5th day. To verify these results, a flow cytometry assay of CD4+ and CD8+ T cells in the distal tumor tissues was performed. The levels of CD4+ and CD8+ T cells, as compared to other groups, were both maximal on the 5th day post-PTT which was consistent with the above results (Fig. 2c, Additional file 1: Fig. S12). Additionally, the quantity of infiltrating natural killer cells (NK cells), macrophages, T-regulatory cells, and mature DCs in distal tumor tissues were investigated by flow cytometry. Infiltration of NK cells, macrophages and mature DCs in the distal tumor was highest on the 5th day after PTT, while it is also concurrent with the lowest level of infiltrating T-regulatory cells. The concurrently low level of infiltrating T-regulatory cells indicated that the anti-tumor immune response in the distal tumors on the 5th day elicited after PTT was the strongest (Additional file 1: Figs. S13–S17).

Cytokines secretion plays a pivotal role in the involvement of immune responses. The changes of various cytokines in the peripheral blood of mice bearing H22 tumors, including TNF-α, IL-6 and IL-12p70, were examined at day 1, 5 and 10 after respective treatments (PBS, surgical resection and PTT) in a parallel experiment. The findings demonstrated that pro-inflammatory cytokines secretion triggered by PTT on the 5th day were greater than any other groups, indicating that the immune microenvironment of the distal tumor tissue is in its most active state on the 5th day post-PTT (Fig. 2d–f).

To determine if temperature would directly affect PD-L1 expression in the tumor cells, the variation in PD-L1 levels was monitored in various types of tumor cells with or without 50 ℃ water-bath treatment. Five types of tumor cells exhibited significant elevation of the mRNA levels of PD-L1 under the 50 ℃ water-bath compared with cells incubated at 37 ℃ (Additional file 1: Fig. S18a, b). This new discovery holds promising potential for immune-checkpoint inhibitor therapy. PD-L1 expression in the distant H22 tumor tissues in the mice under aforementioned treatment was further detected that the mRNA level of PD-L1 expression on the 5th day post PTT was significantly increased (6.33 fold = 5d-PTT/10d-Control), followed by a decline on the 10th day after PTT (1.74 fold) (Additional file 1: Fig. S18c). Consistently, the values obtained on the 5th day were higher than those values on the 10th day in each group. This consistency was also confirmed by Western blot analysis, the immunohistochemistry (IHC) and the quantity of IFN-γ (Additional file 1: Fig. S18d–f). The expression of CRT and HSP70 in tumors at different times after hyperthermia was investigated which revealed that hyperthermia enhanced the expression of CRT and HSP70 significantly on the 5th day in tumor tissues (Additional file 1: Fig. S19).

### CD38 and CD28 expression of CD8+ T cells at different time points post PTT based on PLOV

**Fig. 3:**
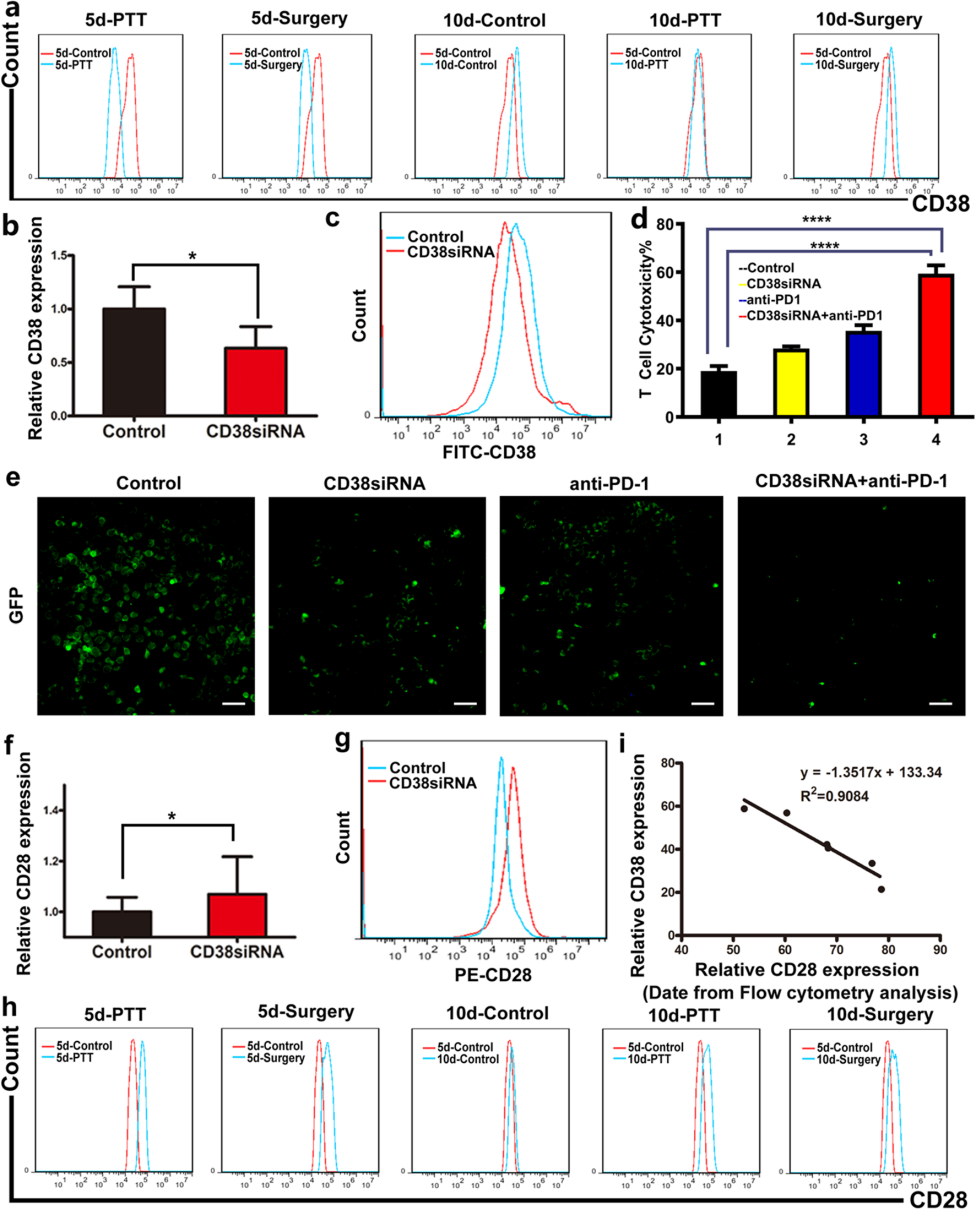
CD38 and CD28 expression in CD8+ T cells at varied time points under different treatment. **a** Flow cytometry analysis of CD38 expression in CD8+ T cells infiltrated in H22 tumor tissue under different treatments as shown in Fig. 2a. **b** The qRT-PCR analysis of CD38 level in CD8+ T cells transfected with CD38siRNAs (50 nM) modified by anti-CD7-9R complex. **c** The flow cytometry assays of T cells transfected with CD38siRNAs (50 nM) modified by anti-CD7-9R complex. **d** Evaluation of T-cell toxicity in 4T1 cell line incubated under different circumstance by LDH dehydrogenase assay. 1 control; 2 CD38siRNA; 3 anti-PD-1; 4 CD38siRNA + anti-PD-1. **e** The assessment of capacity of T cells in killing 4T1 cells under described treatment (CD38siRNA, anti-PD-1, CD38siRNA + anti-PD-1) was represented by intensities of GFP. The scale bar is 50 μm. **f**, **g** Q-PCR analysis and flow cytometry assays were conducted to monitor CD28 level in CD8+ T cells via CD38 blockade. **h** The flow cytometry assays of CD28 expression in CD8+ T cells infiltrated in H22 tumor tissue under PTT or surgery treatments 5 or 10 days. **i** The correlation between CD38 and CD28 expressions in the CD8+ T cells infiltrated in H22 tumor tissue under different treatments. Statistical analysis was conducted by the Student t test for two groups and the one-way ANOVA for multiple groups, and the statistical significance was set as *P < 0.05; ****P < 0.001

To provide further support to the hypothesis that the CD38 expression of CD8+ T cells was closely related to the function of PD-1 antibody, the expression of CD38 in CD8+ T cells was measured under varying treatment conditions as shown in Fig. 2a. As suggested in the flow cytometry analysis (Fig. 3a, Additional file 1: Fig. S20), the quantities of CD38 in CD8+ T cells on the 5th day after PTT (21.41%) was dramatically lower than that in any other groups. This phenomenon was verified by the q-PCR analysis of CD38 expression in T cells in the H22 tumor tissues isolated from the mice with above treatment (Additional file 1: Fig. S21). Additionally, the quantity of CD38 in CD8+ T cells in the spleen isolated from the above mice after varied treatment were then detected by flow cytometry assay to assess the influence of PTT on systematic immunity. By contrast, compared with the obvious variation of CD38 expression in CD8+ T cells induced by PTT in the tumor site, a much smaller reduction in CD38 expression in CD8+ T cells in spleens was observed, suggesting that PTT mainly resulted in the down-regulation of CD38 expression in the newly established tumor immune microenvironment (Additional file 1: Fig. S22). To verify whether CD38 blockading may be a promising strategy for improving the overall efficacy of anti-PD-1 antibody during treatment period, a combination therapy accompanied by the down-regulation of CD38 and anti-PD-1 was conducted to improve anti-tumor immune response. To enhance the transfection efficiency of siRNA into T cells, CD38siRNA was modified with specific CD7 antibodies conjugated with nine arginines as aCD7 specifically targets T cells while nine arginines are positively charged and possess membrane-penetrating properties. The endocytosis of the complex aCD7-9R-siRNA was mediated by the CD7 receptor on the T lymphocyte membrane, proved by the significant reduction in complex uptake by T lymphocytes sealed with CD7 antibodies (Additional file 1: Fig. S23). As demonstrated in q-PCR and flow cytometry assays, depletion of CD38 was successfully performed by CD38siRNA (Fig. 3b, c, Additional file 1: Fig. S24). CD8+ T cells in tumor microenvironment showed more uptake of siRNA and lower CD38 after PTT treatment (Additional file 1: Figs. S25, S26). The expression of CD38 in tumor, myeloid or lymphoid also demonstrated the range of T cell modification was controlled within the tumor without affect other tissues (Additional file 1: Fig. S27). The presence of aCD-7 could significantly affect the expression level of CD38 in T cells (Additional file 1: Fig. S28). To evaluate the capacity of CD38siRNA to modulate cytotoxicity in vitro, 4T1 cell line was screened as target cells to determine cytotoxicity via the LDH release test. As displayed in Fig. 3d, the combination group with CD38 siRNA and anti-PD-1 expressed the greatest cytotoxicity in vitro as compared to anti-PD-1 alone (59.33%), indicating that CD38 blockade might be effective in boosting the therapeutic efficacy of anti-PD-1. To evaluate the function of T cells, the expression of IFN-γ, perforin and granzyme B were determined by ELISA, respectively (Additional file 1: Fig. S29). The highest expression was found within the group co-incubated with anti-PD-1 after CD38 interference. Meanwhile, to explore transfection efficiency of siRNA into T cells by PLOV, T cells were incubated with different formulations for 12 h and 24 h respectively. As demonstrated in q-PCR and flow cytometry assays, depletion of CD38 was successfully performed by siRNA@PLOV while no effect showed in PLOV and scramble@PLOV (Additional file 1: Fig. S30a–c). Simultaneously, the T cell in the combination group with siRNA@PLOV and anti-PD-1 expressed the greatest cytotoxicity in vitro (Additional file 1: Fig. S30d). Furthermore, flow data showed that the expression of CD38 in tumor infiltrating T cells was indeed decreased by siRNA@PLOV (Additional file 1: Fig. S30e, f). The CLSM image exhibited significant colocalization of fluorescence signals with the CD38 (red signals) and FAM-siRNA (green signals) in the tumor tissues (Additional file 1: Fig. S30g). Subsequently, to assess the ability of T cells in killing tumor cells (4T1), Green Fluorescent Protein (GFP) was transfected into 4T1 cells to represent the density of killed 4T1 cells while 4T1 cells under the described treatment (siRNA@PLOV, anti-PD-1, siRNA@PLOV + anti-PD-1) were incubated with T cells. The representative images illustrated that the density of GFP which was minimal in the siRNA@PLOV + anti-PD-1 group was noticeably lower than siRNA@PLOV group and anti-PD-1 group (Fig. 3e). Accordingly, these results identified that combination group with CD38 siRNA and anti-PD-1 could achieve the maximum cytotoxicity.

CD28, as an important costimulatory molecule, plays a vital role in the effective activation and proliferation of T cells. Increased CD28 expression can contribute to increased immune cytokines released by CD8+ T cells, ultimately leading to stronger anti-tumor effects. Subsequently, the q-PCR analysis and flow cytometry assays were conducted to monitor CD28 level in CD8+ T cells via CD38 blockade, revealing a 1.07 fold (CD38siRNA/Control) elevation of CD28 expression (Fig. 3f). The fluorescence signal of CD28 expression was also strengthened through the down-regulation of CD38 (Fig. 3g, Additional file 1: Fig. S31). Western blotting showed the same results (Additional file 1: Fig. S32). This result suggested that inhibition of CD38 could trigger the up-regulation of CD28. To further validate the correlation between CD38 expression and CD28 level, the proportion of CD28 in CD8+ T cells was investigated under the same treatment as mentioned above. Coincidently, compared with other groups, the CD28 level in CD8+ T cells was at the highest level on the 5th day after PTT (Fig. 3h, Additional file 1: Fig. S33). A q-PCR analysis of CD28 expression in the tumor tissues was performed, and the results displayed that CD28 level was significantly increased at day 5 after PTT, which was higher than other groups (Additional file 1: Fig. S34). Spleens were excised from the mice under a forementioned treatment conditions to measure CD28 levels in CD8+ T cells by flow cytometry assay, allowing for monitoring of the systematic change of CD28 expression induced by PTT. Consequently, flow cytometry analysis in the spleens revealed a relatively small increase of CD28 in CD8+ T cells, indicating that PTT mainly induce the alteration of CD28 expression in tumor sites (Additional file 1: Fig. S35). Furthermore, the expression of CD38 and CD28 was linearly fit on the basic of analyzing the above flow analysis results. It was discovered that there was an approximate negative relationship between CD38 and CD28 expression, indicating that the decline of CD38 is accompanied by an elevation in CD28 levels (Fig. 3i).

### siRNA@PLOV-based PTT plus anti-PD-1 to inhibit lung metastasis in fluc-4T1 primary tumor model

**Fig. 4:**
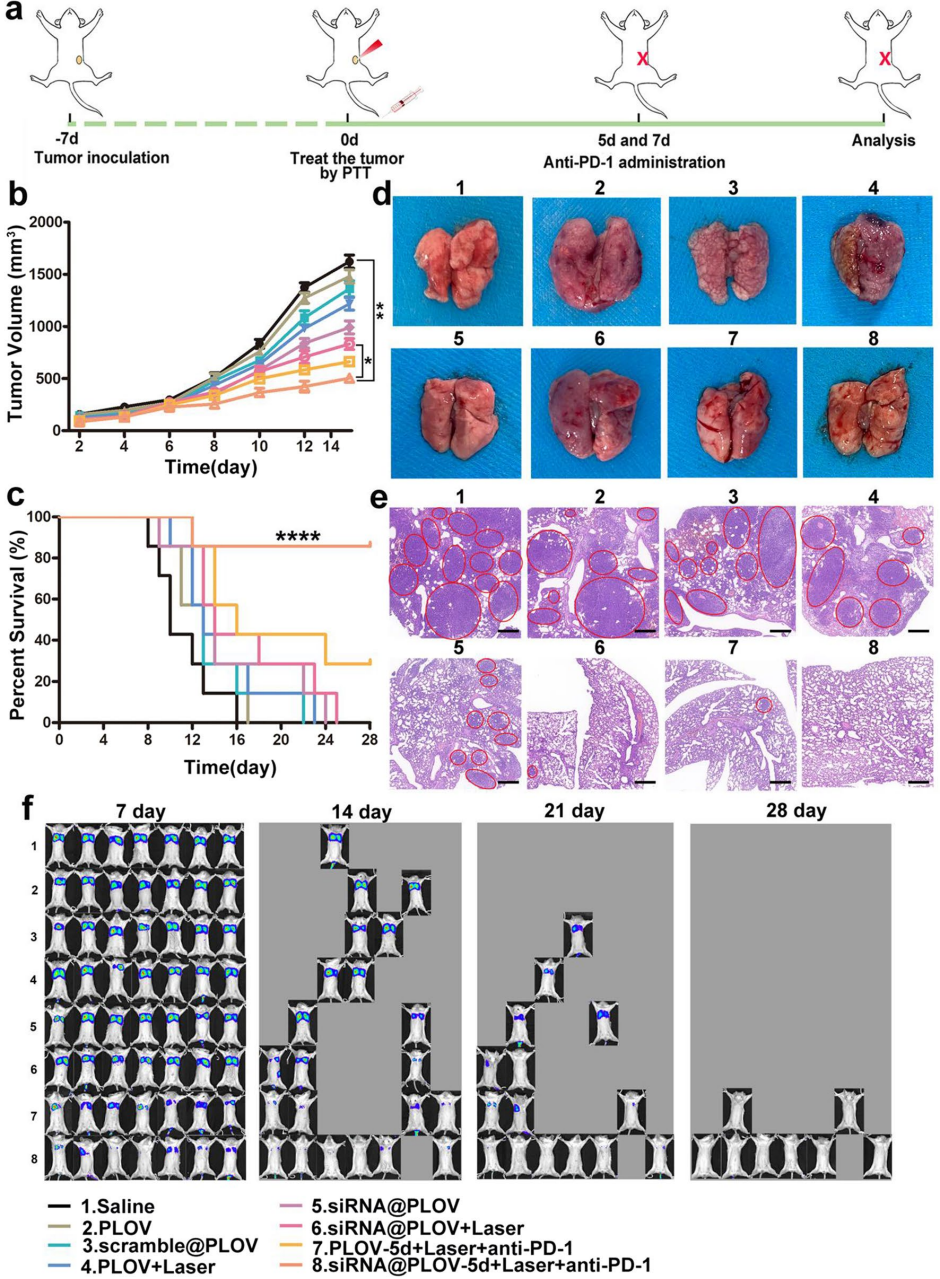
siRNA@PLOV-based PTT combined with anti-PD-1 to suppress lung metastasis after eliminating 4T1 primary tumor. **a** Schematic of siRNA@PLOV-based PTT plus anti-PD-1 to suppress lung metastasis in fLuc-4T1 primary model. **b** The tumor volume of mice (N = 7) with inoculation of fLuc-4T1 orthotopic tumor after receiving different treatments. **c** The survival curve of mice in the fLuc-4T1 orthotopic tumor model with different treatment regimens. **d** The lungs excised from mice in fLuc-4T1 orthotopic tumor model to evaluate the inhibitory effect on pulmonary metastasis. **e** The H&E staining of tumor tissues isolated from above mice. The tumor areas were marked by red circles. The scale bar is 400 μm. **f** Morbidity-free survival of different groups of mice with metastatic fLuc-4T1 tumors in after various treatments indicated to eliminate their primary tumors. Data are given as mean ± SD, N = 7. Statistical analysis was conducted by the one-way ANOVA for multiple groups, and the statistical significance was set as *P < 0.05; **P < 0.01; ****P < 0.001

To testify the therapeutic efficacy of siRNA@PLOV-based PTT plus anti-PD-1 in inhibiting metastasis from primary breast cancer, the metastatic model of fLuc-4T1 orthotopic tumor was introduced in this research. The establishment of animal model and administration method are depicted in Fig. 4a. The pharmacodynamic of infused anti-PD-1 antibody three times was evaluated by estimating PD-1 occupancy on circulating T cells over time. Standard pharmacodynamics measurements of anti-PD-1 serum concentrations yielded an approximate a mean serum peak occupancy of 85% and a mean plateau occupancy of 78%, respectively (Additional file 1: Fig. S36). As illustrated in Fig. 4b, compared with these groups, the combination of siRNA@PLOV-based PTT with anti-PD-1 administration on 5th day exerted stronger inhibition on the tumor growth. Next, the life-span of above mice with various treatments was observed within 35 days (Fig. 4c), and the results revealed that the life-span of mice in siRNA@PLOV-5d + laser + anti-PD-1 group was prolonged to a maximal extent, which was much longer than any other groups. For the other groups of mice with saline alone, PLOV-5d based PTT combined of anti-PD-1 or even the combination of siRNA@PLOV-5d and anti-PD-1 significant bioluminescence signals, indication of tumor metastases, also showed up, although at later stages. To further explore the treatment results of various therapeutic regimen in inhibiting pulmonary metastasis caused by breast cancer, the lungs in mice were excised and the H&E staining of tumor tissues isolated from above mice was also performed (Fig. 4d, e). The results revealed that there was nearly no visible pulmonary metastasis in the lung of mice treated with siRNA@PLOV-based PTT plus anti-PD-1 administration on the 5th day, which was remarkably different from frequent occurrence of metastasis in any other group. The H&E staining results coincided with the pulmonary metastasis which was in line with above results. While mice treated with PLOV-5d based PTT to ablate their primary tumors showed delayed metastases, the group with siRNA@PLOV-5d based PTT together with anti-PD-1 blockade therapy showed nearly no metastasis (Fig. 4f).

Herein, the potential of photothermal combined with enhanced immunotherapy (the immunogenicity of OMVs and CD38 inhibition) via siRNA@PLOV in supplying a new strategy for achieving better curative results of metastatic cancer was considered. As depicted in Additional file 1: Fig. S37a, mice in the siRNA@PLOV-5d + laser + anti-PD-1 group displayed an apparent reduction in tumor volume, demonstrating that siRNA@PLOV-based PTT plus anti-PD-1 on the 5th day exerted strongest repression on the growth of distal tumor (Additional file 1: Fig. S37b). The median survival rates of mice in siRNA@PLOV-5d + laser + anti-PD-1 group was highest in the H22 model, which remained 80% even on the 35th day after treatment (Additional file 1: Fig. S37c). Again, parallel experiments were developed in CT26 models, and the overall tendencies of tumor volume and survival curve were both similar with the measured results in H22 model (Additional file 1: Fig. S37d, e).

These results showed that the treatment effect of anti-PD-1 antibody was maximized by administrating on the 5th day after siRNA@PLOV-based PTT. Furthermore, the mice treated by siRNA@PLOV-based PTT showed the highest levels of all cytokines (IL-6, IL-12p70 and TNF-α) (Additional file 1: Fig. S38).

### siRNA@PLOV-based PTT plus anti-PD-1 to trigger “tumor vaccine” effects

**Fig. 5:**
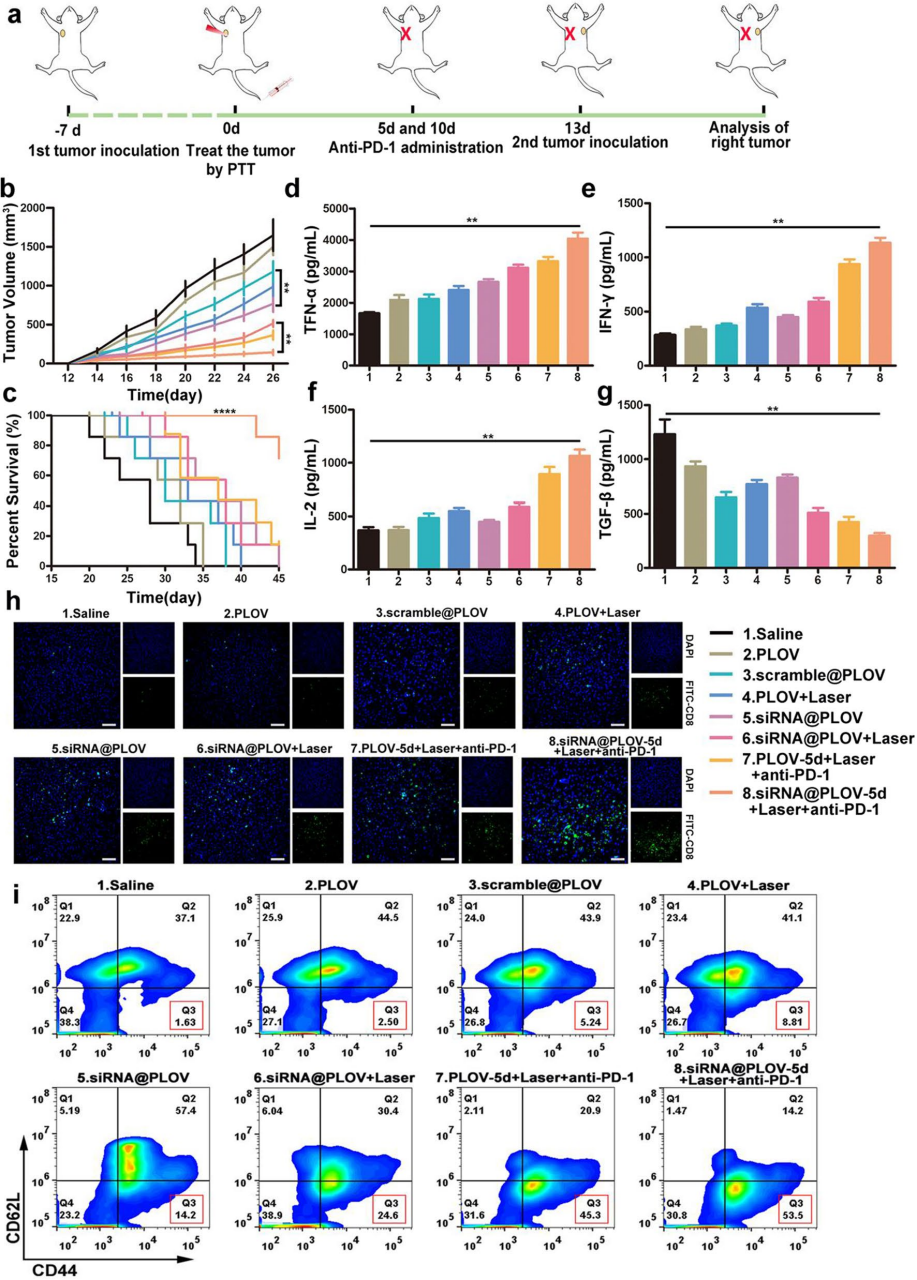
The “tumor vaccine” effect induced by the treatment of siRNA@PLOV-based PTT plus PD1 antibody. **a** The mice with the subcutaneous inoculation of the secondary H22 tumors after eliminating primary tumors by PTT plus anti-PD-1. **b** The tumor volume of mice (N = 7) with subcutaneous inoculation of the secondary H22 tumors after eliminating primary tumors with different approaches. **c** The survival curve of mice with the subcutaneous inoculation of the secondary H22 tumors after eliminating primary tumors with five treatment regimens as described. **d**–**g** The cytokine levels, including TNF-α (**d**), IFN-γ (**e**), IL-2 (**f**) and TGF-β (**g**) in the peripheral blood of mice under different treatments measured by ELISA. Data are given as mean ± SD (n = 7). **h** Immunohistochemical analysis of lymphocyte infiltration in tumor tissue. Green for FITC-CD8, and blue for nucleus. The scale bar is 50 μm. **i** Flow cytometry of memory CD8+ T cells in tumor tissue. Statistical analysis was conducted by the one-way ANOVA for multiple groups, and the statistical significance was set as **P < 0.01; ****P < 0.001

It is of great significance to assess the “tumor vaccine” effect induced by the treatment of siRNA@PLOV-based PTT plus anti-PD-1 antibody. The establishment of animal model and administration method are depicted in Fig. 5a. As revealed in Fig. 5b, mice with their primary tumors eliminated by siRNA@PLOV-based PTT and anti-PD1 administration on the 5th day exhibited dramatic attenuation on tumor growth of secondary H22 tumors, which obtained superior results in comparison to the other groups. The assessment of survival rates was conformed to the previous experimental outcomes (Fig. 5c). To further understand the “tumor vaccine” effect generated by photothermal tumor ablation, some experiments have been proceeded steadily to determine the concentrations of immune factors in blood on the 12th day after the ablation of the primary tumors with different treatments. Consequently, according to the analysis of measured cytokine levels, it was found that examined levels of these four types of cytokines in peripheral blood appeared to be highest in mice treated by combination therapy with siRNA@PLOV-based PTT and anti-PD-1 administration on the 5th day, compared to other groups (Fig. 5d–g). The combination of PTT and aPD-1 significantly promoted tumor immune infiltration of CD8+ T cells, activated DC cells and regulated several types of immunocytes (Fig. 5h, Additional file 1: Figs. S39–S43). The tumor immune environment also revealed high T memory cells after the second tumor vaccination (Fig. 5i), demonstrating the memory immunity evoked by the tumor vaccine. Therefore, it can be concluded that the combination of siRNA@PLOV-based PTT and anti-PD-1 administration on the 5th day may stimulate in vivo “tumor vaccine” effect to the greatest extent.

## Discussion

Heightening the response rate in immune checkpoint therapies remains an essential aspect in cancer treatment, requiring further exploration and advances. Although immune checkpoint therapy utilizing PD-1/PD-L1 antibody has achieved largely comforting clinical therapeutic results in the battle against cancer, such therapy is available only to a limited minority [37, 38]. The efficacy of PD-1 antibody, however, is closely associated with differences between the early and late-stage tumor immune microenvironment [39]. Alteration of CD38 expression in CD8+ T cells in the immune microenvironment as a result of immune checkpoint blockading (anti-PD-1 antibody) influences significant differences across treatments in both early and late-stage tumors. The failure of anti-PD-1 antibody may stem from the elevated CD38 expression in the long-established tumor immune microenvironment. The hybrid fusion vesicle inherits and potentiates the immunological functions of the OMVs ingredients and photothermal ability of photothermal sensitive liposomes, conferring not only an efficient DC-based immunoactivation, but also a powerful PTT which enhanced infiltration of immune cells. Therefore, it was hypothesized that the late-stage tumor immune microenvironment can be rescued by reconstructing a newly established tumor immune environment via PLOV-PTT bearing similar characteristics of an early-stage tumor microenvironment. PLOV-PTT stimulated maturation of DCs and infiltration of CD4+ and CD8+ T cells in tumor tissues was observed, revealing distinctly higher infiltration of tumor-infiltrating lymphocytes on the 5th day as compared to the 10th day post-PTT. Most notably, the 5th day post-PTT experienced substantial downregulation of CD38 expression on CD8+ T cells, confirming the previous hypothesis.

Emerging as a promising target for improvement of anti-PD-1 therapeutic benefits, CD38 has gained notoriety due to its upregulated expression in the late-stage tumors. In vitro and in vivo experiments revealed that blockading CD38 could precipitously augment the treatment efficacy of anti-PD-1 although the underlying mechanism remained unclear. While some researches have concluded that there is a negative correlation between CD38 and CD28 expressions in T cells, CD28, as a co-stimulator of T cells, could significantly enhance the killing capacity of CD8+ T cells [40]. It was hypothesized that the improvement of immune checkpoint therapy due to reduction in CD38 levels may be involved with the concurrent enhancement of CD28 expression. Data revealed that the inhibition of CD38 trigger the upregulation of CD28, effectively rescuing dysfunctional CD8+ T cells and improving the therapeutic effect of the PD-1 antibody.

Immunotherapy is a modern means of cancer treatment which has achieved superior therapeutic effect. However, the concurrent excess immune response stimulates detrimental systematic side. To avoid triggering unnecessary immunity in whole body scale, which causes T cells to attack normal tissues, a cascaded double-target biomimetic nanosystem was constructed to directly target tumor-infiltrating T-cell in tumor tissues. The primary aim of the constructed nanosystems was in limiting the accumulation of carriers at the tumor sites. Reduction of CD38 expression in spleens modulated by the cascaded double-target biomimetic nanosystem-based PTT was relatively smaller than that in tumor tissues, so did the myeloid and lymphoid. This variance indicated that such a multifunctional drug delivery system is capable of effectively avoiding the occurrence of excess immune response.

## Conclusions

While the combination of hyperthermia with immunotherapy has been widely employed in cancer therapy, the mechanism of its unique synergistic effect has not been studied thoroughly. In the present study, hyperthermal treatment based on biomimetic nano system effectively reshaped the tumor immune microenvironment, thereby allowing late-stage tumor tissues to return to the earlier, active stage of tumor development. Mechanically, the PLOV is capable to initiate the anticancer immune response owing to the self-adjuvating OMVs fused. Results demonstrated that the immune environment of tumor tissues was most conducive to the combined immunotherapeutic approach 5 days after hyperthermia. In addition, the expression of PD-L1 in tumor cells, a dynamic stress response, was effectively stimulated by higher temperature and INF-γ. Simultaneously, CD38 was determined an effective target which can be used to enhance the efficacy of immune checkpoint inhibitors. The cascaded targeting nanosystem siRNA@PLOV constructed in this study can organically combine hyperthermia and self-adjuvant with CD38 inhibition. When combined with the PD-1 antibody, it showed good therapeutic effect and low toxic side effects in both mice tumor metastasis model and vaccine treatment model. Given that all components utilized in the formation of the drug delivery system are safe, biodegradable reagents, it possesses great potential for clinical transformation. Therefore, this study will provide ideas and serve as future research basis for the improvement of the therapeutic effects of tumor immune checkpoints.

## Materials and methods

### Animals

All animal experiments were carried out in compliance with the Animal Management Rules of the Ministry of Health of the People’s Republic of China (document NO. 55, 2001) and all research involving animals strictly complied with protocols approved by the Animal Welfare and Ethics Committee (AWEC, China Pharmaceutical University) and the number is 2021-06-003. The license number for the use of laboratory animals is SYXK(SU)2016-0011. All animals used in the experiment were ~ 8-week-old BALB/c mice and ICR mice purchased from Qinglongshan Animal Feeding Center, Nanjing. Both males and females were used for the experiments reported in this study.

### Bacteria culture and cell cultures

All cell lines used in the experiment were purchased from American Type Culture Collection (ATCC, Shanghai). CT26 (mouse colon cancer cell; ATCC:TCM37), H22 (mouse hepatocellular carcinoma cell; ATCC:CC-Y2040), 4T1 (mouse breast cancer cell; ATCC:TCM-32), HepG2 (human hepatocellular carcinoma cell, ATCC:TCHu 72), MCF7 (human breast cancer cell; ATCC:TCHu 74), U-87 MG (human brain astroblastoma, ATCC:TCHu138) and A549 (human non-small cell lung cancer cell; ATCC:TCHu 150). All cells were cultured in Dulbecco’s modified Eagle medium (DMEM) containing 10% fetal bovine serum (FBS), 100 IU/mL of penicillin, and 100 μg/mL Cells were maintained at 37 °C in a humidified atmosphere with 5% CO_2_. Unless otherwise specified, cells were harvested by scraping in ice-cold PBS and pelleting by centrifugation at 1200 rpm for 5 min.

BMDCs were removed from marrow cavities of femurs and tibias of mice and cultivating in the lower chamber of transwell plates with DMEM containing 10 ng/mL GM-CSF and 5 ng/mL IL-4 for 7 days.

Attenuated *Salmonella* were purchased from American Type Culture Collection (ATCC, Rockville MD). Attenuated *Salmonella* were used as the sources of outer membrane vesicles (OMVs). Attenuated Salmonella were cultured on Luria broth (LB) medium. All research involving OMVs strictly complied with protocols approved by the MISEV 2018.

### Preparation of siRNA@PLOV

Outer membrane vesicles (OMVs) were obtained from 250 mL bacteria LB medium at late exponential-phase about protein concentration 1 μg/μL. NTA was performed to detect the concentration of obtained OMVs which was 10^6^ particles/mL. The concentration of nanoparticles in the in vitro experiment is 10^3^ particles/mL, and the concentration in vivo experiment is 10^6^ particles/mL. Briefly, 250 mL medium was centrifuged 15 min at 4000×*g* to remove attenuated *Salmonella* and then the supernatant was filtered through 0.45 μm pore size filters (Millipore, USA). The filtrate was further concentrated by centrifugal filters at 14,000×*g*. The OMVs were collected and washed twice with PBS at 4 °C for 1.5 h by ultracentrifugation at 150,000×*g* and stored at PBS. The photosensitive liposomes (PTSLs) incorporated with Cypate were first prepared at a mass ratio of 34.3512:8.4816:1 (DPPC:DPPG:Cypate). The phospholipid 1,2-dihexadecanoyl-snglycero-3-phosphocholine (DPPC) and 1,2-dipaimitoylsn-glycerol-3-phospho-(1′-rac-glycerol) (DPPG) were purchased from A.V.T (Shanghai, China). And then the lipid film was formulated by rotary evaporation at 55 ℃, 150 mbar. The hydration of the lipid film was performed by 5 mL PBS to carry on rotary evaporation at 50 ℃ under atmospheric pressure for 2 h. The resultant solution was sonicated at 50 ℃ for 20 min. Then the resultant PTSLs and OMVs were transferred to mini-extruder and co-extruded. The mixture of scFvCD7-9R-siRNA were loaded into PLOV to form siRNA@PLOV by ultrasonic method.

### Physicochemical characterization of PLOV

All the hydrodynamic size and zeta potential were measured by using a Malvern ZS90 zeta-sizer as shown in previous publications. The size of OMVs, PTSLs and PLOV were measured by a JEM-2000 EX II transmission scanning electron microscope (TEM, JEOL Company, USA).

### SDS-PAGE and western blot protein analysis

SDS-PAGE was employed to analyze the proteins. The PTSLs, OMVs and PLOV were prepared in loading buffer as measured by BCA kit. The samples were heated to 95 °C and kept for 5 min and 30 μg of the sample was loaded into each well of an 8% SDS polyacrylamide gel. The samples were run at 80 V for 40 min and 110 V for 90 min, and then the resulting polyacrylamide gel was stained with Coomassie blue for 2 h, washed overnight, and then visualized. FijB and SipC antibody were used to detect OMV and PLOV by western blot protein analysis.

### Two-step co-incubation transwell

Firstly, 4T1 cells are cultured in the upper chamber and BMDCs are seeded in the lower chamber. Different formations of nanoparticles (siRNA@PTSL, siRNA@OMV, siRNA@PLOV) are added into the co-incubated cells with or without NIR irradiation to evaluate the immune stimulation toward BMDCs. Then suspended BMDCs in PBS with anti-mouse antibody against CD80-FITC, and CD86-PE. Similar processing was applied in in the 4T1 solid tumor model. For quantitative release of cytokines, the DC medium supernatants were measured by ELISA. T cells were labeled at 37 ℃ with 4 μmol/L CFSE for 10 min. A total of 1 × 10^5^ CFSE-labeled T cells were incubated with DCs in transwell system for 72 h in the upper chamber, and the proliferation of T cells was analyzed by flow cytometry.

### Immunofluorescence assay to evaluate CD8+ T cells in distal tumor tissues of different treatment groups

For the inoculation on the bilateral sides of each female ICR mouse (N = 7), the inoculation was performed by subcutaneous injection of H22 (2 × 10^5^) cell suspension on bilateral sides of each mouse. After 1 week, the primary tumors of mice were treated by PTT via the intravenous injection of PLOV or surgery. The distal tumor tissues were isolated from mice in 5th day or 10th day after treatment. The collected tumors were placed in 4% paraformaldehyde for 6 to 8 h and transferred to 20% sucrose and the frozen tissue pieces were embedded. A frozen tissue section of 8 μm thick was prepared by a cryostat fixed with acetone for 10 min, and stored at − 20℃ for immunofluorescence. The above sections were washed three times with PBS solution for 3 to 5 min each time. Subsequently, the sections were then blocked with 2% BSA at room temperature for 1 h. After incubation with CD8+ primary antibody (eBioscience) overnight at 4 ℃, the slices were washed three times with PBS. Subsequently, it was incubated with a secondary antibody at room temperature for 1 h, and washed with PBS. After staining with Hoechst (keygen) for 10 min, it was washed twice with PBS, an anti-quenching agent was added dropwise to seal, and it observed using a confocal scanning laser microscope (CSLM) (Olympus, FV1000).

### Analysis of different T cells after various treatments detected by flow cytometry

For analysis of CD4+ and CD8+ T cells, CD28 expression in CD8+ T cells infiltrated and various cytokines in the distal tumor tissues of mice, the inoculation was performed by subcutaneous injection of H22 (2 × 10^5^) cell suspension on bilateral sides of each female ICR mouse (N = 7). After 1 week, the primary tumors of mice were treated by PTT via the intravenous injection of PLOV or surgery. Tumor drainage lymph nodes were harvested from mice in 5th day or 10th day after treatment and ground with the rubber head of a syringe. Cells were filtered through nylon mesh filters and washed with PBS. After adding lymph extract, the cells were centrifuged by gradient centrifugation to obtain a complete lymphocyte suspension. The complete lymphocyte suspension (200 μL) was further stained with anti-CD8-FITC (eBioscience), anti-CD4-FITC (eBioscience), anti-Foxp3-PerCP, anti-CD28-PE (eBioscience) and anti-CD38-FITC (eBioscience) at 0.25 μg/test for 20 min. The cell suspensions mentioned above were collected for data analysis by flow cytometry (BD Biosciences, America).

### Cytokine examination

The levels of tumor necrosis factor (TNF-α, Dakewe biotech), IL-6 (Dakewe biotech), and IL-12p70 in the distal tumor tissues of mice bearing H22 tumors were examined at day 1, day 5 and day 10 after respective treatment (PBS, surgical resection and PTT). The tumor homogenates were determined by ELISA kits.

The peripheral blood samples of mice after different approaches were collected with 100 μL EDTA·2Na at the concentration of 15 mg/mL. Then, plasma was obtained by centrifugation at 3000 rpm for 15 min for analysis of several cytokines, including TNF-α, IFN-γ, IL-2, and TGF-β by ELISA kits.

Additionally, the expression of IFN-γ, perforin and granzyme B from T cells under varying incubation circumstances were determined by ELISA kits. The T cells were cultured in Roswell Park Memorial Institute-1640 (RPMI-1640) without serum and treated with CD38 siRNA, anti-PD-1 or combination group. After 12 h treatment, 6 mL medium supernatant was absorbed with the centrifugation at 1000*g* for 20 min to perform an ELISA test was then.

### Western blot analysis of PD-L1 expression

Different types of cells were treated in water bath at 50 ℃ or 37 ℃ for 20 min and then cultured for 6 h. The tumor tissues were treated by PTT or surgery and collected on the fifth or tenth day after treatment. Cells and tumor tissues were lysed in ice-cold Lysis buffer containing a protease inhibitor cocktail and 100 mM phenylmethylsulfonylfluoride (PMSF) for 5 min on ice. After repeated freeze–thaw for 3 times, the supernatant was obtained by centrifugation at 20,000*g* for 20 min. The proteins were prepared in loading buffer as measured by BCA kit. The samples were heated to 95 °C. Proteins were separated on a 10% SDS-PAGE and electrophoretically transferred to polyvinylidene fluoride membranes by wet method where it was blocked with 5% skim milk in TBST buffer for 1 h at room temperature. Membranes were incubated with PD-L1 primary antibodies overnight at 4 ℃, followed by incubation with the secondary antibody at room temperature. Ultimately, membranes were scanned and band intensity was quantified by using Quantity One Imaging Software from Bio-Rad.

### Quantitative polymerase chain reaction (q-PCR) assays

The mRNA levels of CD28, CD38, and PD-L1 was measured by ABI 7300 (Applied Biosystem, USA). β-actin served as reference. Total RNA was extracted from tissues according to manufacturer protocols using a total RNA purification kit (Shenergy Biocolor BioScience & Technology Company, Shanghai, China). Using the first strand cDNA synthesis kit (Fermantas, Vilnius, Lithuania), RNA (2 μg) was reverse transcribed to cDNA according to manufacturer protocol. PD-L1 primers were sequenced as: upstream primer: 5ʹ-TACCTCTGGCAC ATCCTC-3ʹ; downstream primer: 5ʹ-GTCCTCCAAATGTGTATC-3ʹ. Murine CD38 q-PCR primers, upstream primer sequence: 5ʹ-TGG TTT CCA TCA GAC ACC GC-3ʹ; downstream primer sequence: 5ʹ-AAAATCCTCCTGGCCCCTTC-3ʹ.

### Immunohistochemistry assay

To examine the PD-L1 expression in the distal tumor tissues under varying treatments, the tumor tissues were isolated from each mouse in different treatment groups to perform immunohistochemistry assay. The collected tumors were placed in 4% paraformaldehyde for 6 to 8 h and transferred to 20% sucrose and the frozen tissue pieces were embedded. A frozen tissue section of 8 μm thick was prepared by a cryostat fixed with acetone for 10 min. The above sections were washed three times with PBS solution for 3 to 5 min each time. Subsequently, the sections were then blocked with 2% BSA at room temperature for 1 h. After incubation with PD-L1 primary antibody overnight at 4 ℃, the slices were washed three times with PBS. Subsequently, it was incubated with a secondary antibody at room temperature for 1 h, and washed with PBS. After staining with Hoechst (keygen) for 10 min, it was washed twice with PBS, an anti-quenching agent was added dropwise to seal, and it observed using a confocal scanning laser microscope (CSLM) (Olympus, FV1000).

### Transfection of CD38 siRNA into T cells by anti-CD7-9R

The fusion of nine arginine residues with neuron cell targeting peptide (9R) enables CD38siRNA (5ʹ-CCAAGAACCCUUGCAACAUTT-3ʹ) to be delivered to neuron cells. The amplified anti-CD7-Cys was cloned into pET 26b (+) vector by plasmid. The recombinant protein was purified and refolded by HPLC. T cells were isolated and purified by sheep erythrocyte method. A 1640 medium containing 10% fetal bovine serum was added. Cell culture dishes were placed in a cell culture box containing 5% CO_2_ at 37 ℃. Before transfection, anti-CD7-9R diluted to 1 μL every 50 μL medium. Diluted CD38 siRNA was mixed with anti-CD7-9R and placed for 20 min to form a CD38 siRNA-anti-CD7-9R complex. T-cell suspension was added into the 6-well plate at the volume of 1 mL per well and divided into a blank group and CD38 siRNA group, respectively. After incubation for 6 h, the complex was removed and replaced with culture medium containing 10% fetal bovine serum.

For flow cytometry analysis of siRNA uptake of T lymphocytes, FAM labeled siRNA was used to construct the complex 9R-siRNA or aCD7-9R-siRNA. For the group “T lymphocyte(aCD7)”, CD7 specific antibodies were used to block CD7 receptors on T lymphocytes. After incubating the antibody for 24 h, the nanocomposite was co-cultured with the cells for 1 h and ingested by flow cytometry.

For flow cytometry analysis of siRNA uptake in different cells, FAM labeled siRNA was used to construct nano system and treated mice in groups. After PTT treatment, tumors were ground to extract various cells. PE-CD3, FITC-CD8 were used to recognize CD8+ T cells; APC-CD44 was used to recognize H22 tumor cells.

For effect of the presence or absence of aCD7 on the expression of CD38 in T cells, after cells were incubated with each group of nanoparticles for 1 h, the medium was removed and the expression of CD38 was detected after 6 h of culture.

### In vitro cytotoxicity assay

To evaluate the capacity of CD38 siRNA to modulate cytotoxicity of T cells in vitro, T cell line was co-incubated with 4T1 tumor cells at a mix ratio of 20:1. The 4T1 cells were stimulated with hyperthermia (50 ℃ water-bath) to promote the antigen exposure. Subsequently, the cells were incubated at 37 ℃ and 5% CO_2_ for 8 h. The cell culture plates were centrifuged at 400*g* for 5 min and 120 μL supernatant of each well was taken to a new 96 well plates. Then, 60 μL LDH detection solution added to each well and incubated at room temperature for 30 min in dark. Finally, OD values were obtained by Microplate Reader at 490 nm wavelength.

### Histological analysis

To further explore the therapeutic effects of tail vein injection of liposome or saline in 4T1 tumor-bearing mice. The mice with intravenous injection of saline served as the control group. Subsequently, the tumor tissues were isolated for histopathological analysis on the 13th day post-injection. The obtained tissues were fixed with 10% neutral buffered formalin and embedded in paraffin. The lung cross-sections (8 mm) were stained with hematoxylin and eosin (H&E) and observed with a BX41 bright field microscope (Olympus).

### In vivo assays

For the inoculation on the bilateral sides of each female BALB/c mouse (N = 7), the inoculation was performed by subcutaneous injection of 4T1 (2 × 10^5^) or CT26 (4 × 10^5^) cell suspension on bilateral sides of each mouse. The inoculated tumor of the left side served as the primary tumor, while the tumor of the right side functioned as an artificial mimic of tumor metastasis. After 1 week, the primary tumors of mice were treated by PTT via the intravenous injection of various carriers (saline, siRNA@PLOV, PLOV). The mice were intravenously injected with anti-PD-1 antibody at doses of 200 μg per mouse three times on day 1 and 2 from the 5th or 10th day after PTT, respectively. The corresponding analysis was performed on the 13th day after PTT-based treatment. The tumor volume was calculated based on the following formula: width^2^ × length × 0.5.

The cell suspensions of fLuc-4T1 (2 × 10^5^) were inoculated into the breast pad of each mouse to establish a fLuc-4T1 lung metastases cancer model. Mice were injected with the relevant substrate before bioluminescence imaging, which was carried out using an in vivo imaging instruments (IVIS) spectrum system with 60 s exposure time. Besides, after 1 week, the tumor was eliminated by PTT via the intravenous injection of various carriers (saline, siRNA@PLOV, PLOV), the day of PTT was chosen as zero point. Next, the mice were intravenously injected with anti-PD-1 antibody at doses of 200 μg per mouse three times on day 1 and 2 from the 5th or 10th day after PTT. On day 7, 14, 21, and 28 after PTT treatment, IVIS imaging was performed to investigate tumor metastasis in the lungs of mice. The lung was excised to analyze the pulmonary metastasis three times for each mouse.

To study the “tumor vaccine” effects triggered by siRNA@PLOV-based PTT plus anti-PD1. The primary tumors were performed by subcutaneous injection of H22 (4 × 10^5^) cell suspension on bilateral sides of each mouse. After 1 week, the tumor was eliminated by PTT via the intravenous injection of various carriers (saline, siRNA@PLOV, PLOV), the day of PTT was chosen as zero point. Next, the mice were administrated with anti-PD-1 antibody (200 μg per mouse) in 5th and 10th day. Then, the mice were re-inoculated with H22 tumor at the contralateral flank, the growth of the secondary tumor was recorded. All mice were sacrificed when the size of the right tumors in the saline group exceeded 2 cm^3^. Frozen sections of dissected tumors were labeled with FITC-CD8 and DAPI to demonstrate immune invasion. After Grind the tumor into a single cell suspension, CD8+ T cells were cycled by flow cytometry and the CD44+ CD62L− cell ratio of memory T cells was analyzed. DC cells in single cell suspension were sorted by CD45+ CD11c+, and the percentage of CD80 and CD86 double-positive DC cells was analyzed by flow cytometry. CD3+ T cells, NK cells, Tregs, M1 macrophages were analyzed by flow cytometry. For all PTT treatment in all tumor models, tail-vein injection with several groups. The mice in the laser treatment groups were subjected to laser exposure (150 mW/cm^2^ for 10 min) at 4 h post-intravenous injection.

### Statistical analysis

Statistical analysis was conducted by the Student’s t test for comparison of two groups and one-way ANOVA for multiple groups A. P value of < 0.05 was considered significant (*), while a P value of < 0.01 was considered very significant (**).

## Supplementary Information

**Additional file 1. **Additional figures S1–S43.

## Data Availability

The datasets in the current study are included in the published article or available from the corresponding author on reasonable request.

## Abbreviations

PTSLs: Photothermal sensitive liposomes
OMVs: Outer membrane vesicles
PD-1: Programmed cell death protein 1
NADase: NAD+ glycohydrolase
PTT: Photothermal therapy
CRT: The translocation of calreticulin
HSP: Heat shock proteins
9R: Nona-d-arginine peptide
CLSM: Confocal laser scanning microscopy
ELISA: Enzyme-linked immunosorbent assay
CTLs: Cytotoxic T lymphocytes
NK cells: Natural killer cells
IHC: Immunohistochemistry
CT26: Mouse colon cancer cell
H22: Mouse hepatocellular carcinoma cell
4T1: Mouse breast cancer cell
HepG2: Human hepatocellular carcinoma cell
MCF7: Human breast cancer cell
U-87 MG: Human brain astroblastoma
A549: Human non-small cell lung cancer cell
DMEM: Dulbecco’s modified Eagle medium
FBS: Fetal bovine serum
LB: Luria broth medium
DPPC: Phospholipid 1,2-dihexadecanoyl-snglycero-3-phosphocholine
DPPG: 1,2-Dipaimitoylsn-glycerol-3-phospho-(1′-rac-glycerol)
TEM: Transmission scanning electron microscope
TNF-α: Tumor necrosis factor-α
RPMI-1640: Roswell Park Memorial Institute-1640
PMSF: Phenylmethylsulfonylfluoride
q-PCR: Quantitative polymerase chain reaction
H&E: Hematoxylin and eosin
IVIS: In vivo imaging instruments
