# Supplementary material for: A cascade targeting strategy based on modified bacterial vesicles for enhancing cancer immunotherapy
Source: J Nanobiotechnology. 2021 Dec 20;19:434. doi: 10.1186/s12951-021-01193-9 (PMC8686283; doi:10.1186/s12951-021-01193-9)
Supplement: Supplementary file 1 — Additional file 1. Additional figures S1–S43. [file 12951_2021_1193_MOESM1_ESM.docx]

**Additional information**

**A cascade targeting strategy based on modified bacterial vesicles for enhancing cancer immunotherapy**

Yuewen Zhai^a,1^, Yuying Ma^a,1^, Bo Pang^d,1^, Jinnan Zhang^b^, Ying Li^a^ , Yalan Rui^a^, Tian Xu^a^, Yu Zhao^d^, Zhiyu Qian^c^, Yueqing Gu^a,^*, Siwen Li^a,^*

*a. State Key Laboratory of Natural Medicines, Jiangsu Key Laboratory of Drug Screening, Department of Biomedical Engineering, School of Engineering,* *China Pharmaceutical University,* *No. 639 Longmian Avenue, Jiangning District, Nanjing 211198, China.*

b. *Department of Neurosurgery, China-Japan union Hospital, Jilin University, Changchun, Jilin, China.*

c. *Department of Biomedical Engineering, School of Automation, Nanjing University of Aeronautics and Astronautics,* *29th JiangJun street, Nanjing 211106, Jiangsu Province, China.*

d. *Jilin Ginseng Academy, Changchun University of Chinese Medicine, Changchun 130117, China*

1. These authors contributed equally and should be regarded as co-first authors.

*.These authors should be co-corresponding authors.

E-mail: [lsw@cpu.edu.cn](mailto:lsw@cpu.edu.cn) [gusubmit@163.com](mailto:gusubmit@163.com).

**Figure S1**


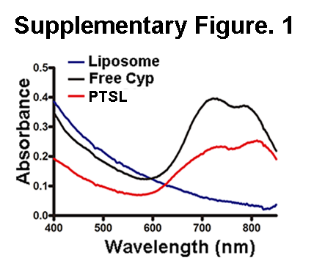


Figure S1. The absorption spectra of free Cyp, Liposomes and PTSL.

**Figure S2**


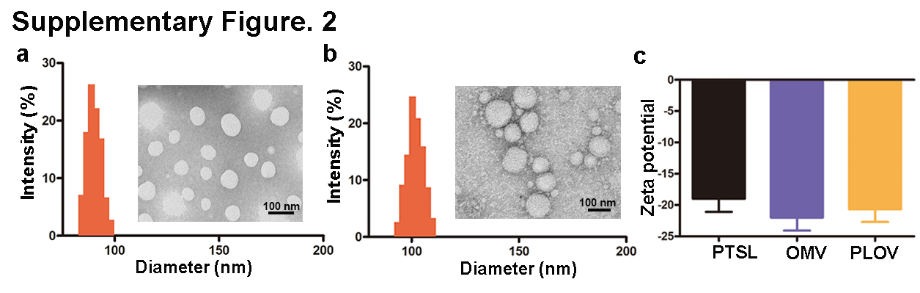


Figure S2. **Characterization of nano system.** (a-b) the TEM image and the hydrodynamic diameter of PTSL (a) and OMVs (b). (c) the variation of zeta-potentials of nanosystems during its layer coating process.

**Figure S3**


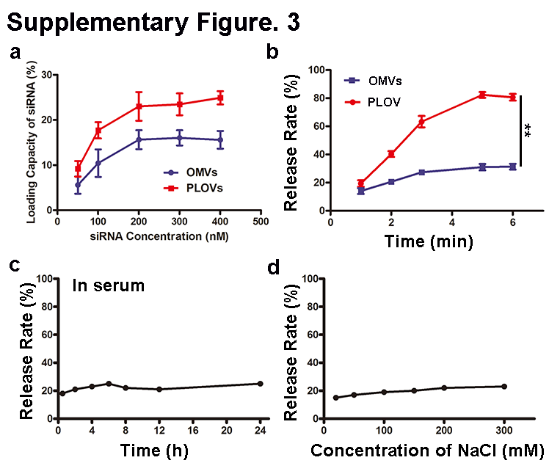


Figure S3. (a) the DL of siRNA in carriers. (b, c, d) The release rates of siRNA from PLOV were determined at different circumstance, including different laser irradiation time, OMVs (10^3^particles/mL) (b), serum (c) and various concentrations of NaCl (d), etc. Statistical analysis was conducted by the student t test, and the statistical significance was set as **P < 0.01, n = 5.

**Figure S4**


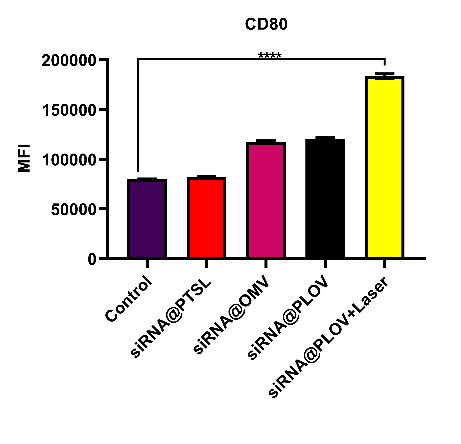

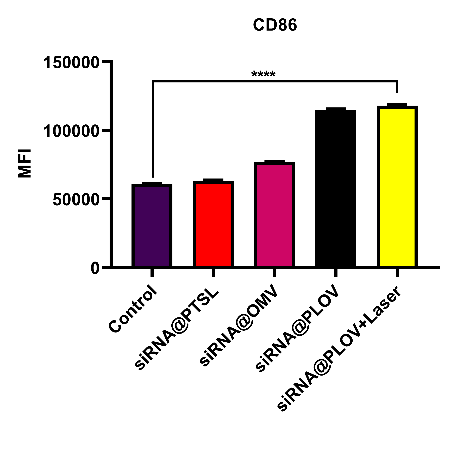


Figure S5. The quantification of CD80 and CD86 expression in DC cells for Figure 1g. Statistical analysis was conducted by the one-way ANOVA for multiple groups, and the statistical significance was set as ****P < 0.001, n = 5.

**Figure S5**


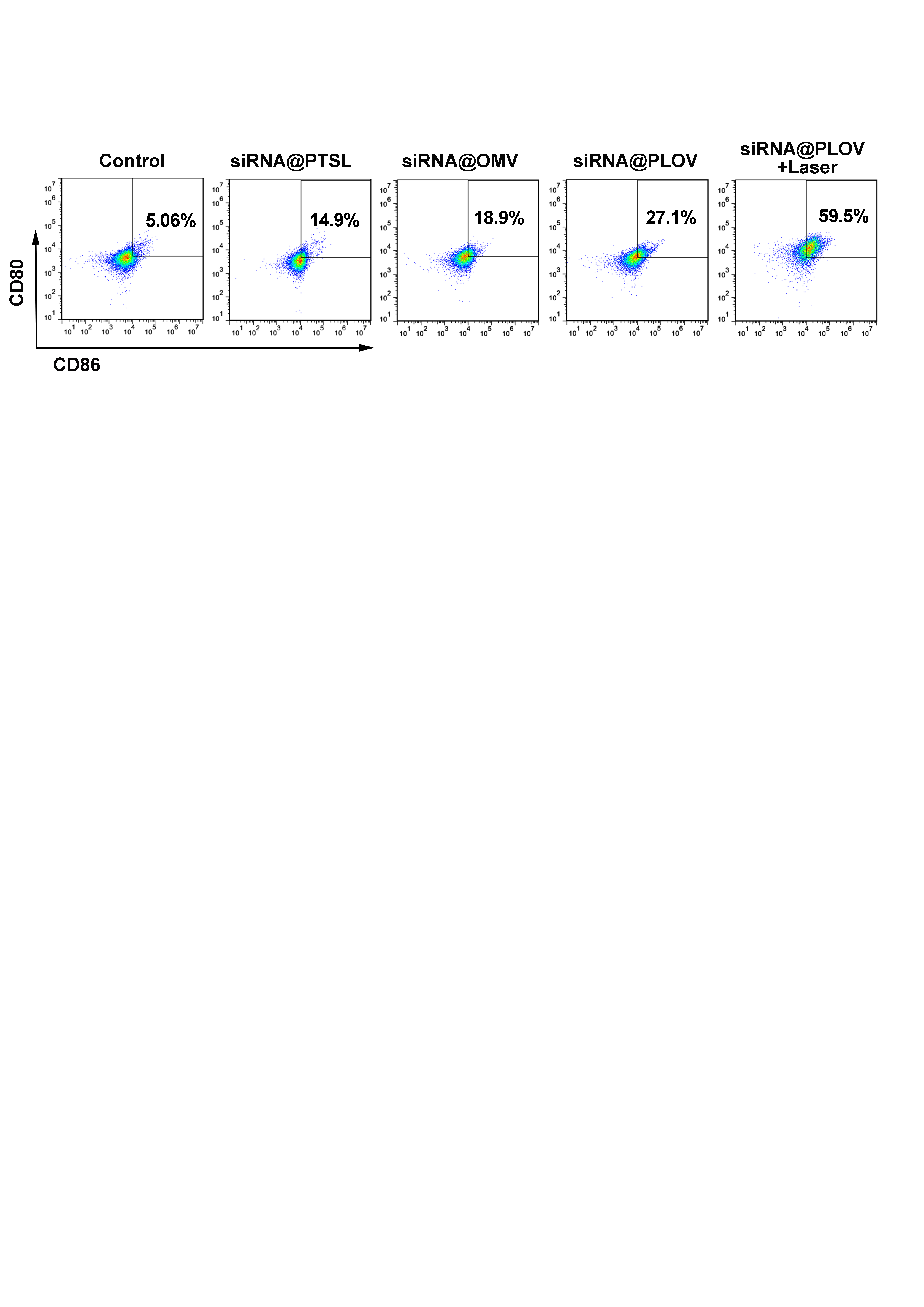


Figure S4. The percentage of CD80 and CD86 double-positive DC cells analyzed by flow cytometry in the 4T1 solid tumor model in vivo. CD80-FITC, and CD86-PE.

**Figure S6**


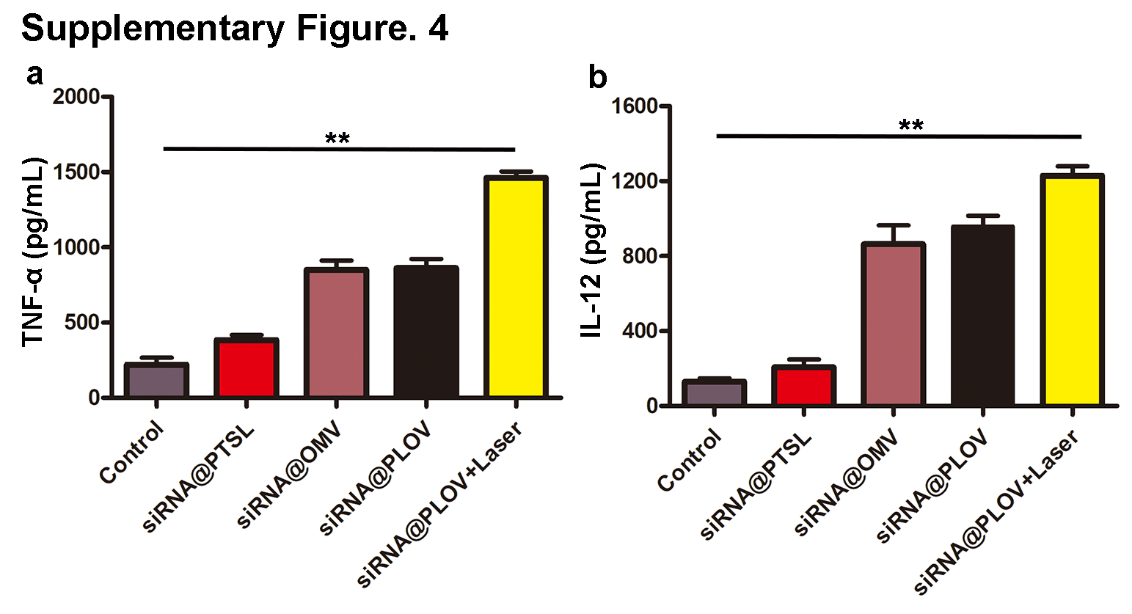


Figure S6. Secretion of TNF-α and IL-12 from BMDCs supernatant under different treatments. Statistical analysis was conducted by the one-way ANOVA for multiple groups, and the statistical significance was set as **P < 0.01, n=5.

**Figure S7**


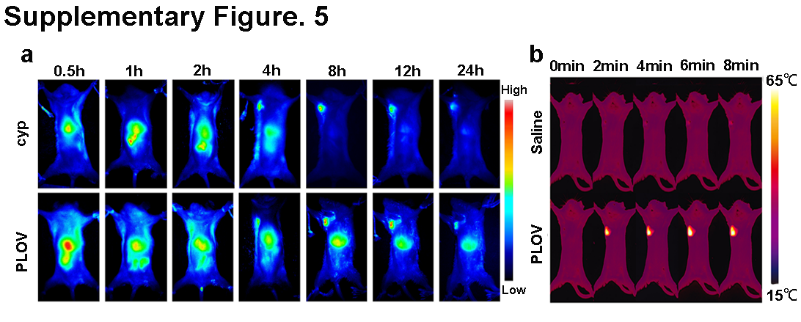


Figure S7. Distribution and photothermal effects in vivo. (a) The near-infrared fluorescence imaging of H22 tumor-bearing nude mice to evaluate the tumor-targeting capacity and bio-distribution of siRNA@PLOV in vivo. (b) The IR thermal images of mice received intratumoral injection of saline and the drug carrier system under irradiation with 808 nm laser (0.5W/cm^2^) once every 2 minutes for 8 minutes.

**Figure S8**


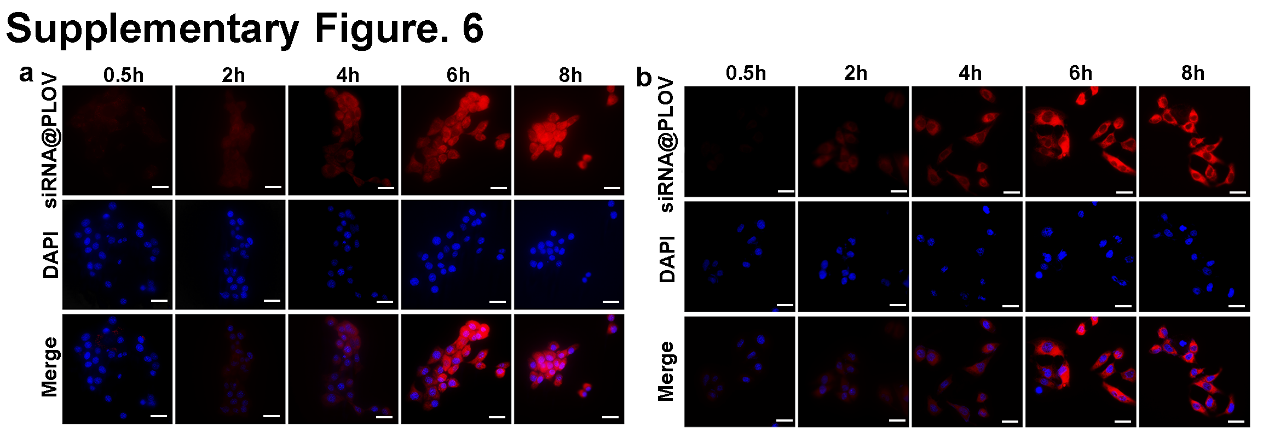


Figure S8. LCSM image of 4T1 cells (a) and HepG2 cells (b) incubated with siRNA@PLOV within 8 h to assess cells uptake capacity. The scale bar is 20 μm.

**Figure S9**


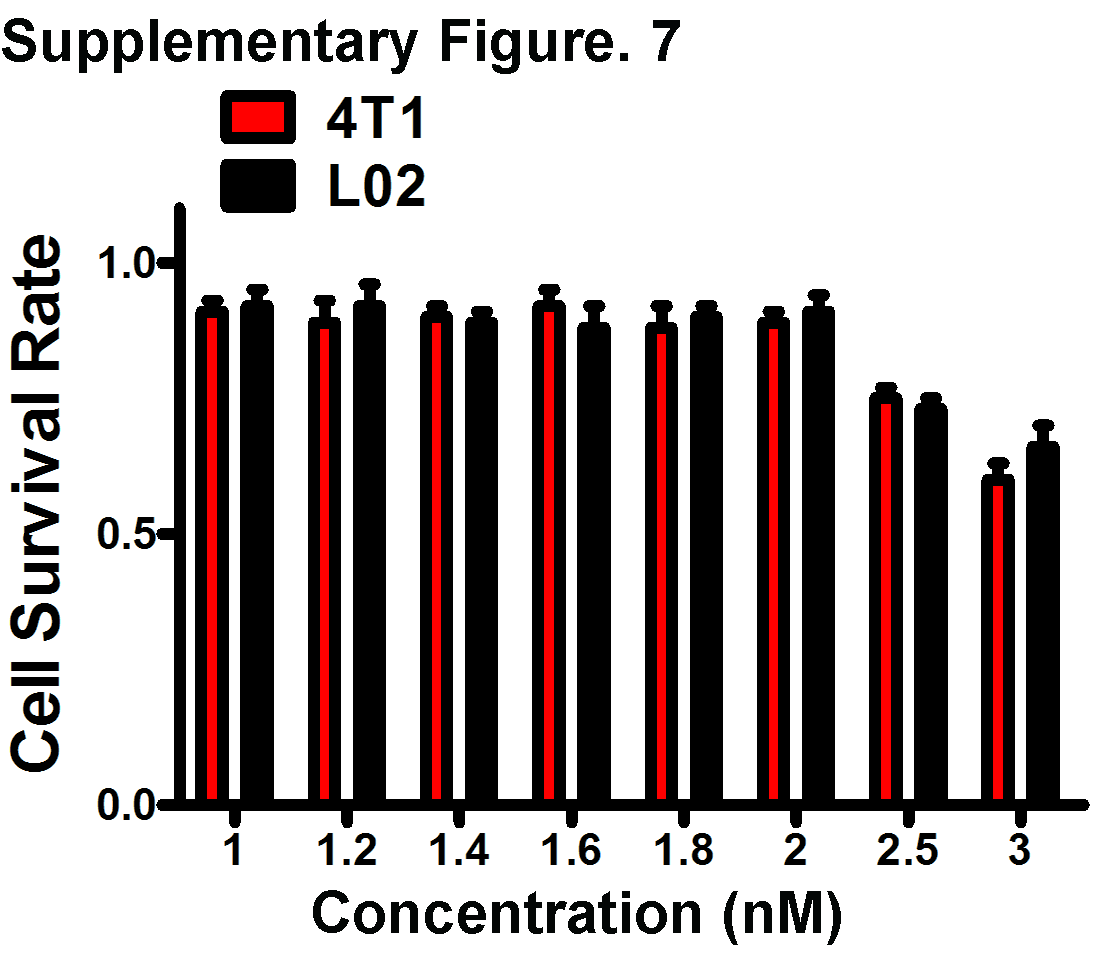


Figure S9. The survival rates of 4T1 and L02 cells incubated with various concentrations of liposomes, n = 5.

**Figure S10**


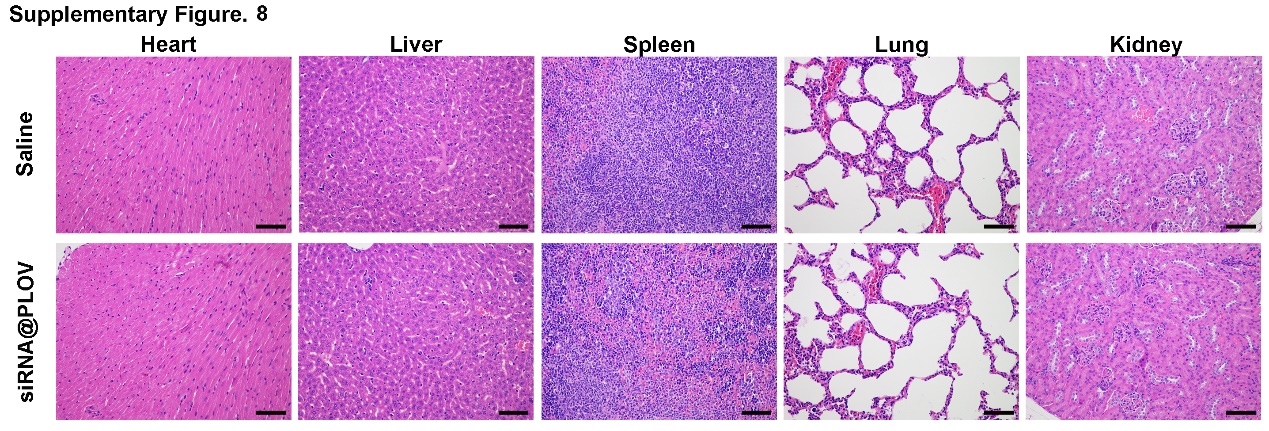


Figure S10. The H&E staining of various organs was performed to test in vivo toxicity of the siRNA@PLOV. The scale bar is 100 μm.

**Figure S11**


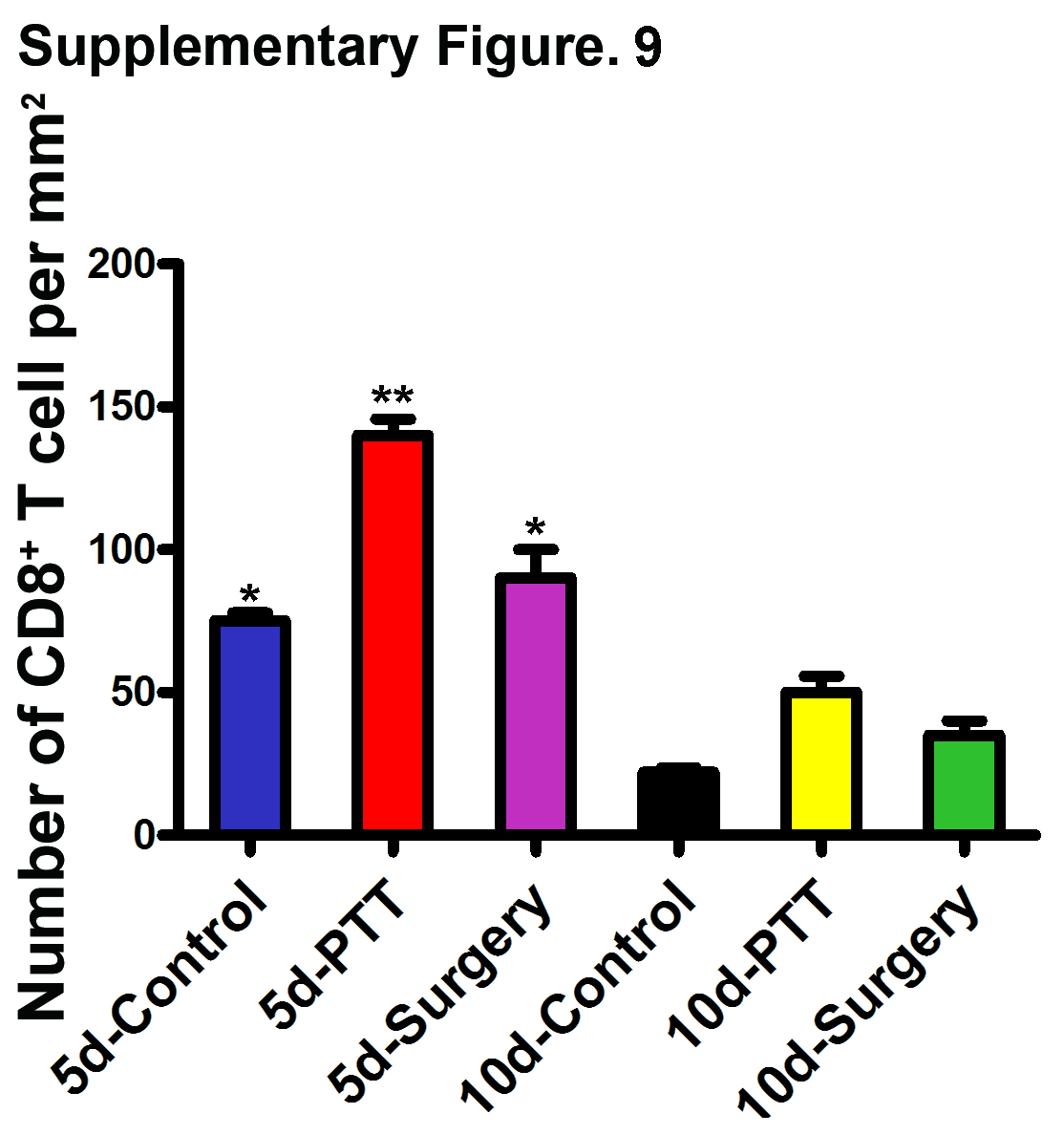


Figure S11. The quantification of CD8+ T cells infiltrated in distal tumor tissues of mice under different treatments. Statistical analysis was conducted by the one-way ANOVA for multiple groups, and the statistical significance was set as *P < 0.05; **P < 0.01, n = 5.

**Figure S12.**


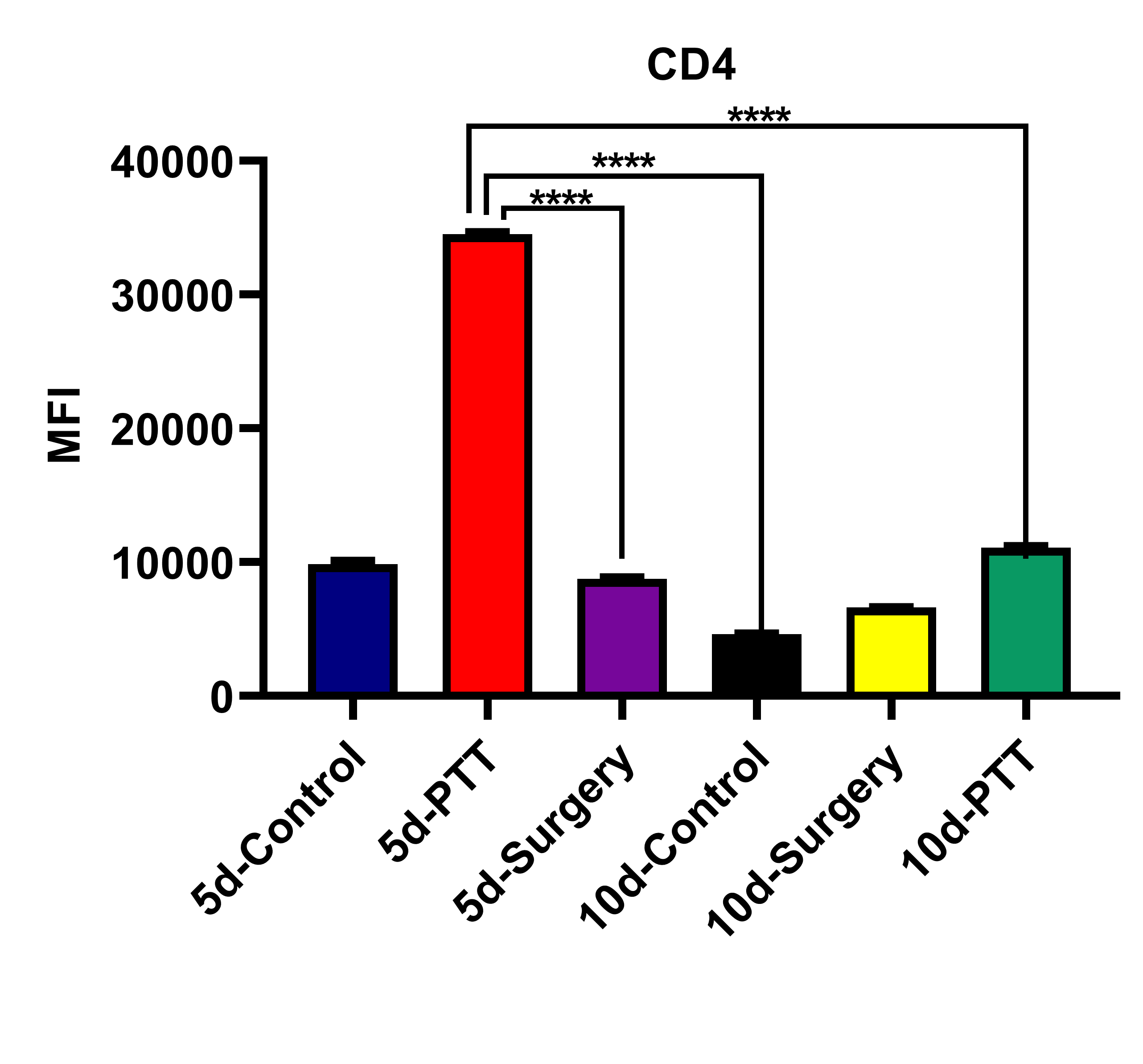

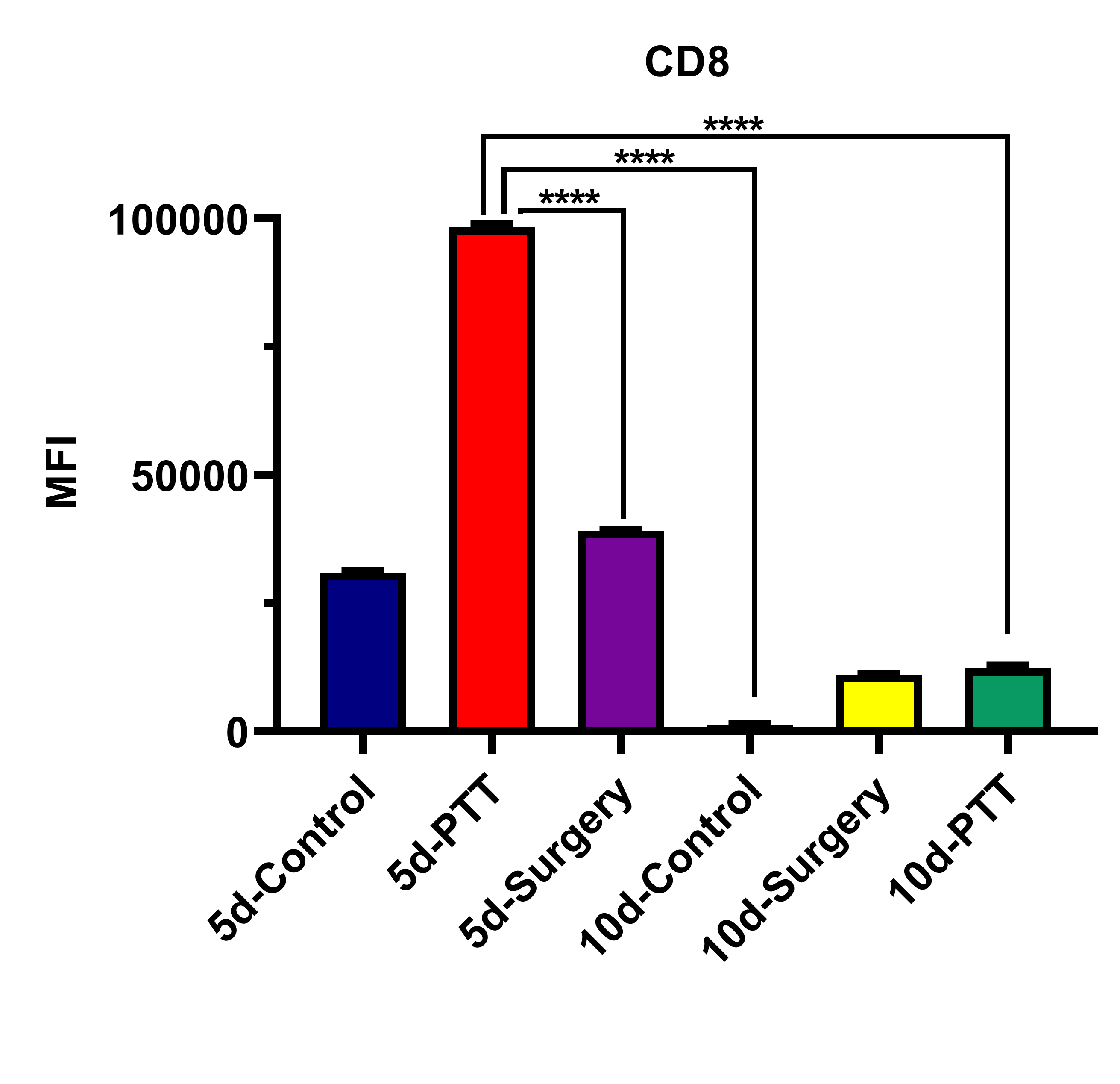


Figure S12. The quantification of CD4+ T cells and CD8+ T cells infiltrated in distal tumor tissues of mice under different treatments for Figure 2c. Statistical analysis was conducted by the one-way ANOVA for multiple groups, and the statistical significance was set as ****P < 0.001, n = 5.

**Figure S13**


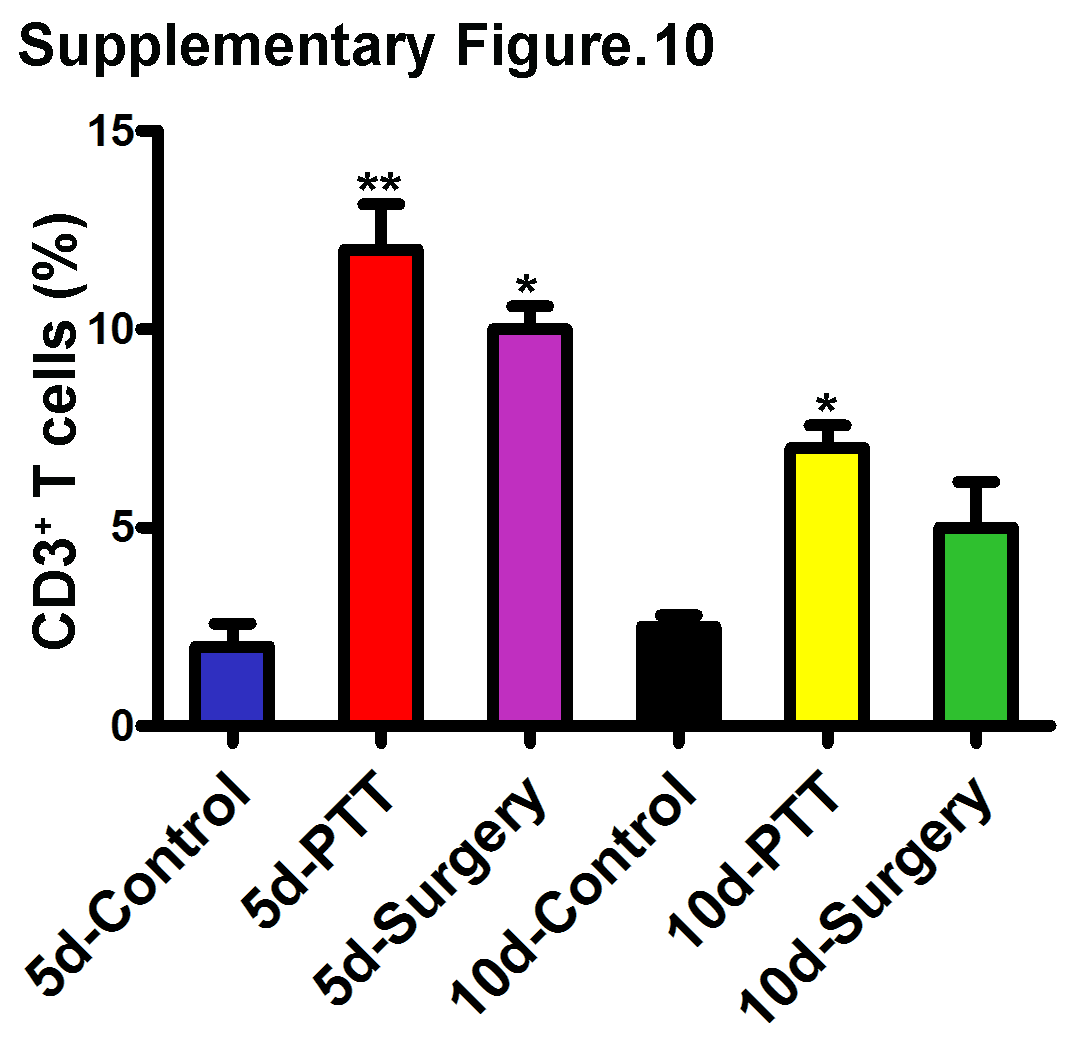


Figure S13. The quantification of CD3+ T cells infiltrated in distal tumor tissues of mice under different treatments. Statistical analysis was conducted by the one-way ANOVA for multiple groups, and the statistical significance was set as *P < 0.05; **P < 0.01, n = 5.

**Figure S14**


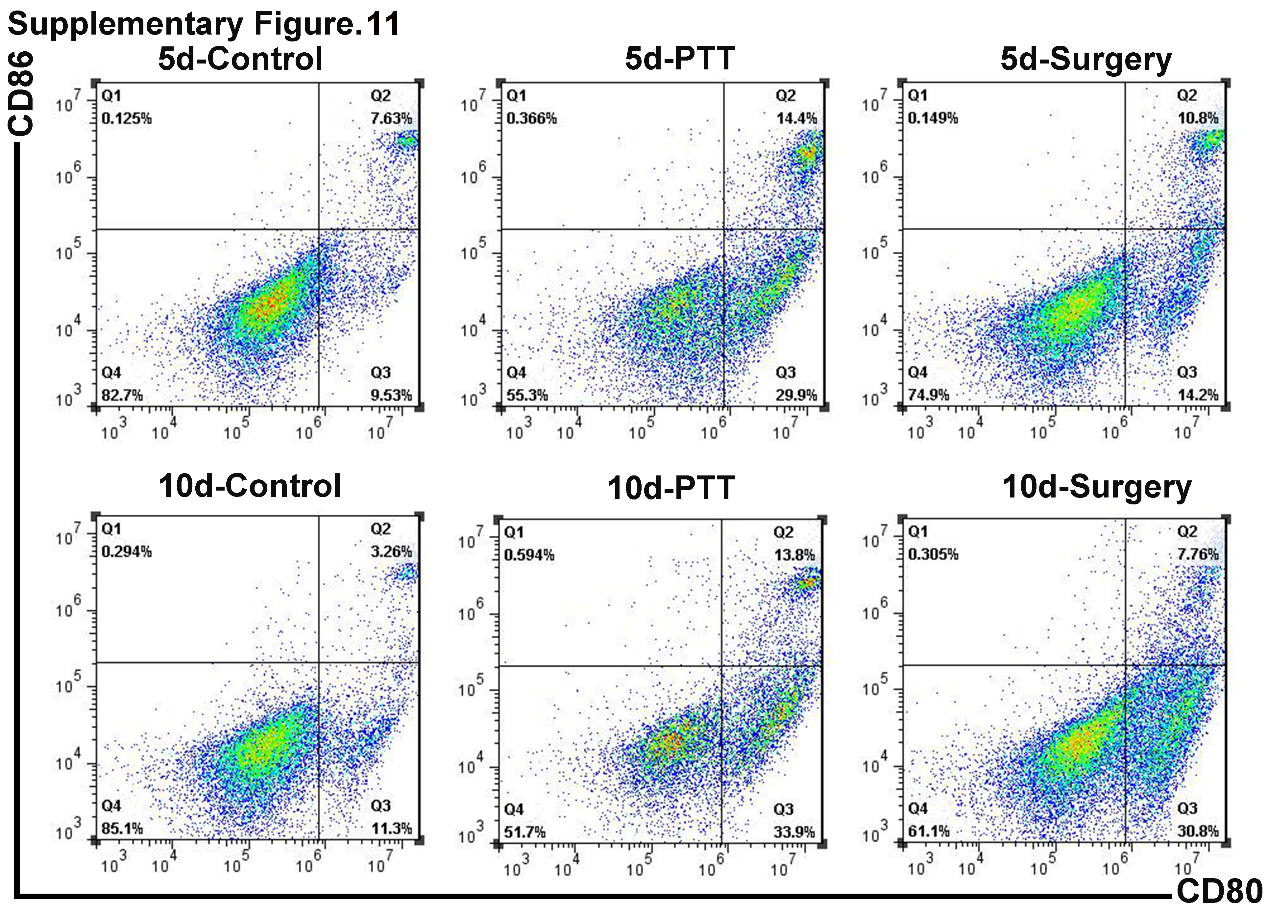


Figure S14. The amounts of **mature DCs** infiltrated in distal tumor tissues of mice under different treatments.

**Figure S15**


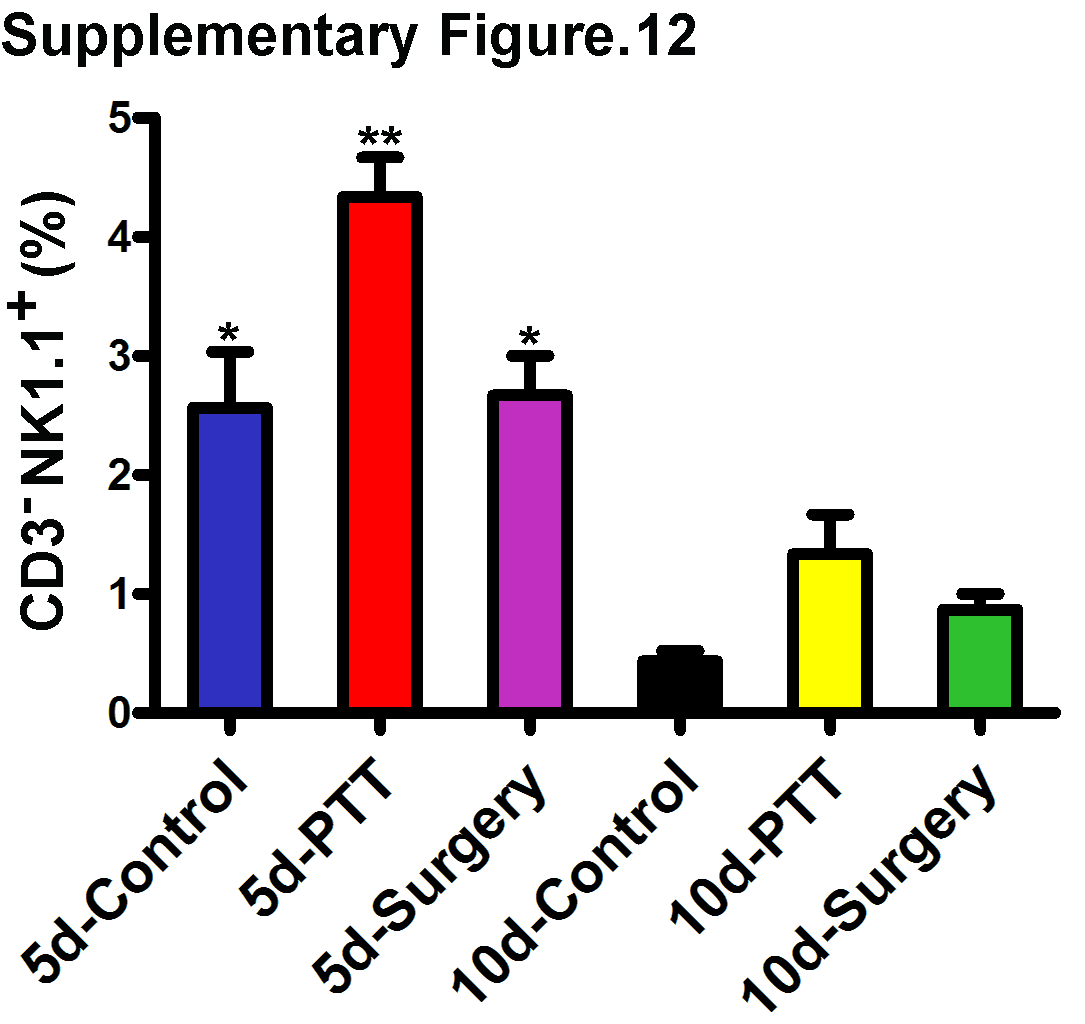


Figure S15. The amounts of **NK cells** infiltrated in distal tumor tissues of mice under different treatments. Statistical analysis was conducted by the one-way ANOVA for multiple groups, and the statistical significance was set as *P < 0.05; **P < 0.01, n = 5.

**Figure S16**


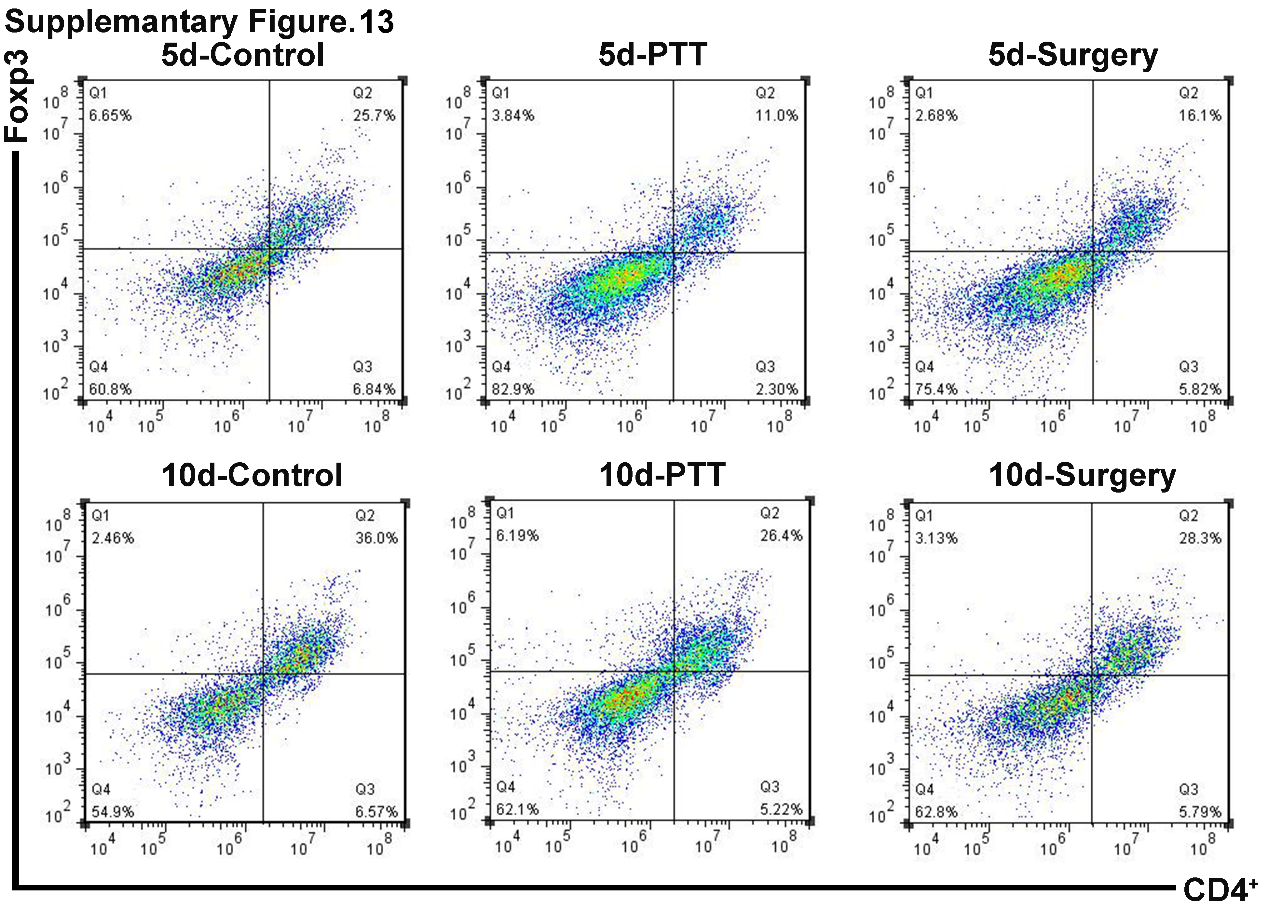


Figure S16. The levels of **Tregs** infiltrated in distal tumor tissues of mice under different treatments were examined by flow cytometry assays.

**Figure S17**


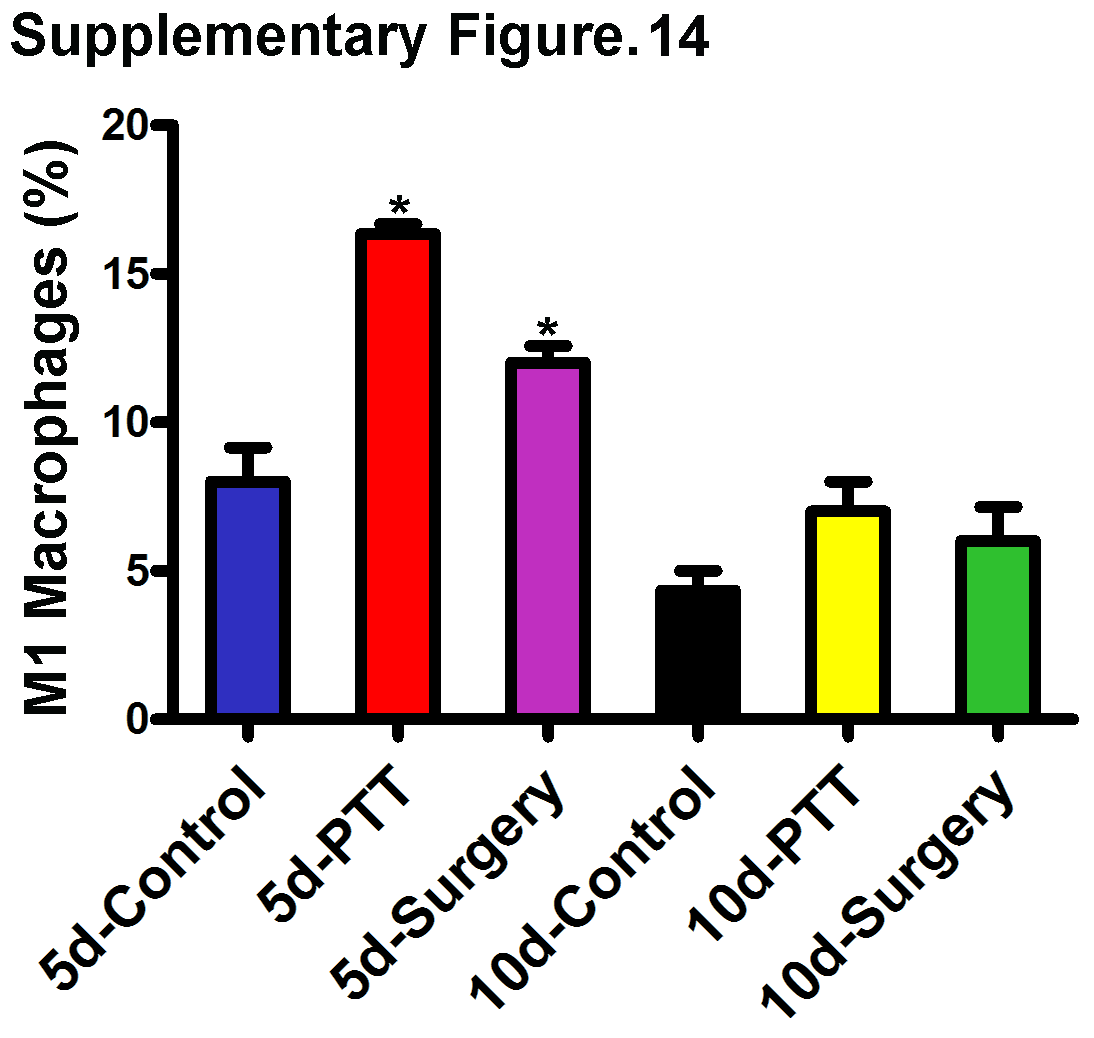


Figure S17. The levels of **M1 Macrophages cells** infiltrated in distal tumor tissues of mice under different treatments. Statistical analysis was conducted by the one-way ANOVA for multiple groups, and the statistical significance was set as *P < 0.05, n = 5.

**Figure S18**


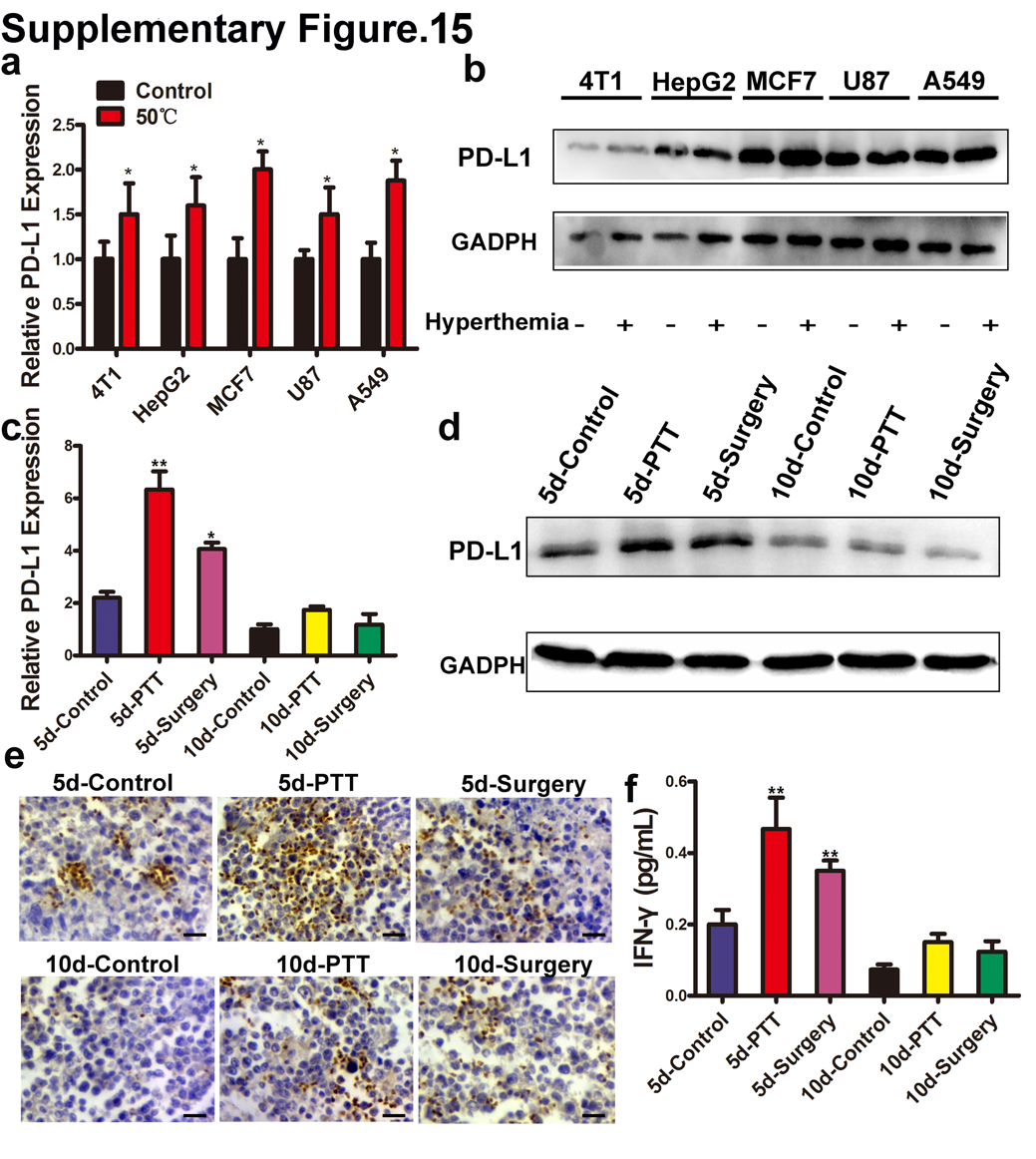


Figure S18. **Influence of hyperthermia on the PD-L1 expression in tumor tissues at different time points.** (a) The q-PCR analysis of PD-L1 expression in various types of tumor cells lines with or without hyperthermia treatment (50℃ water-bath). (b) PD-L1 expression in various cell lines (4T1, HepG2, MCF7, U87 and A549) with hyperthermia treatment (50℃ water-bath) monitored by western blot assays. The cell lines incubated at 37℃ were served as corresponding control groups, respectively. (c) The mRNA levels of PD-L1 in the distant H22 tumor tissues of mice under different treatments detected by q-PCR analysis. (d) The PD-L1 expression in H22 tumor tissues under the varied treatment determined by western blot analysis. (e) The PD-L1 expression of H22 tumor tissues isolated from different groups was examined by IHC. The scale bar is 20 μm. (f) The quantities of IFN-γ with different treatments were detected in peripheral blood of H22 tumor-bearing mice by ELISA. Statistical analysis was conducted by the one-way ANOVA for multiple groups, and the statistical significance was set as *P < 0.05 **P＜0.01, n = 5.

**Figure S19**


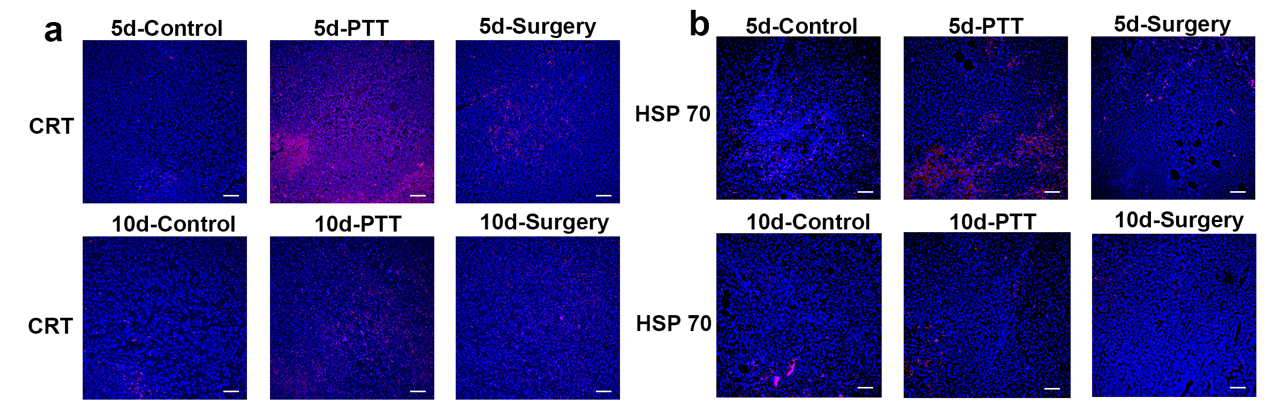


Figure S19. Immunofluorescence of CRT and HSP70 in tumor tissues of different treatment groups. The scale bar is 100 μm.

**Figure S20**


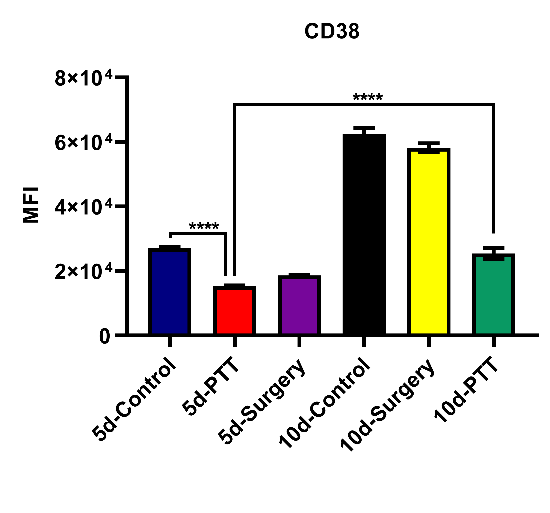


Figure S20. The quantification of flow cytometry analysis of CD38 expression in CD8+ T cells infiltrated in H22 tumor tissue under different treatments for Figure 3a. Statistical analysis was conducted by the one-way ANOVA for multiple groups, and the statistical significance was set as ****P < 0.001, n = 5.

**Figure S21**


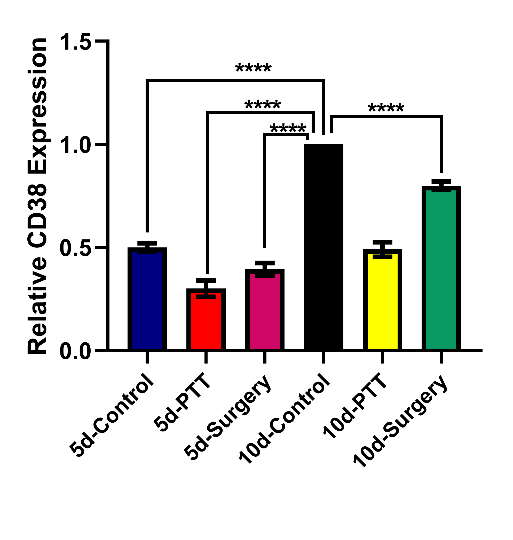


Figure S21. The q-PCR analysis of CD38 expression in T cells in the H22 tumor tissues isolated from the mice with above described treatments. Statistical analysis was conducted by the one-way ANOVA for multiple groups, and the statistical significance was set as ****P < 0.001, n = 5.

**Figure S22**


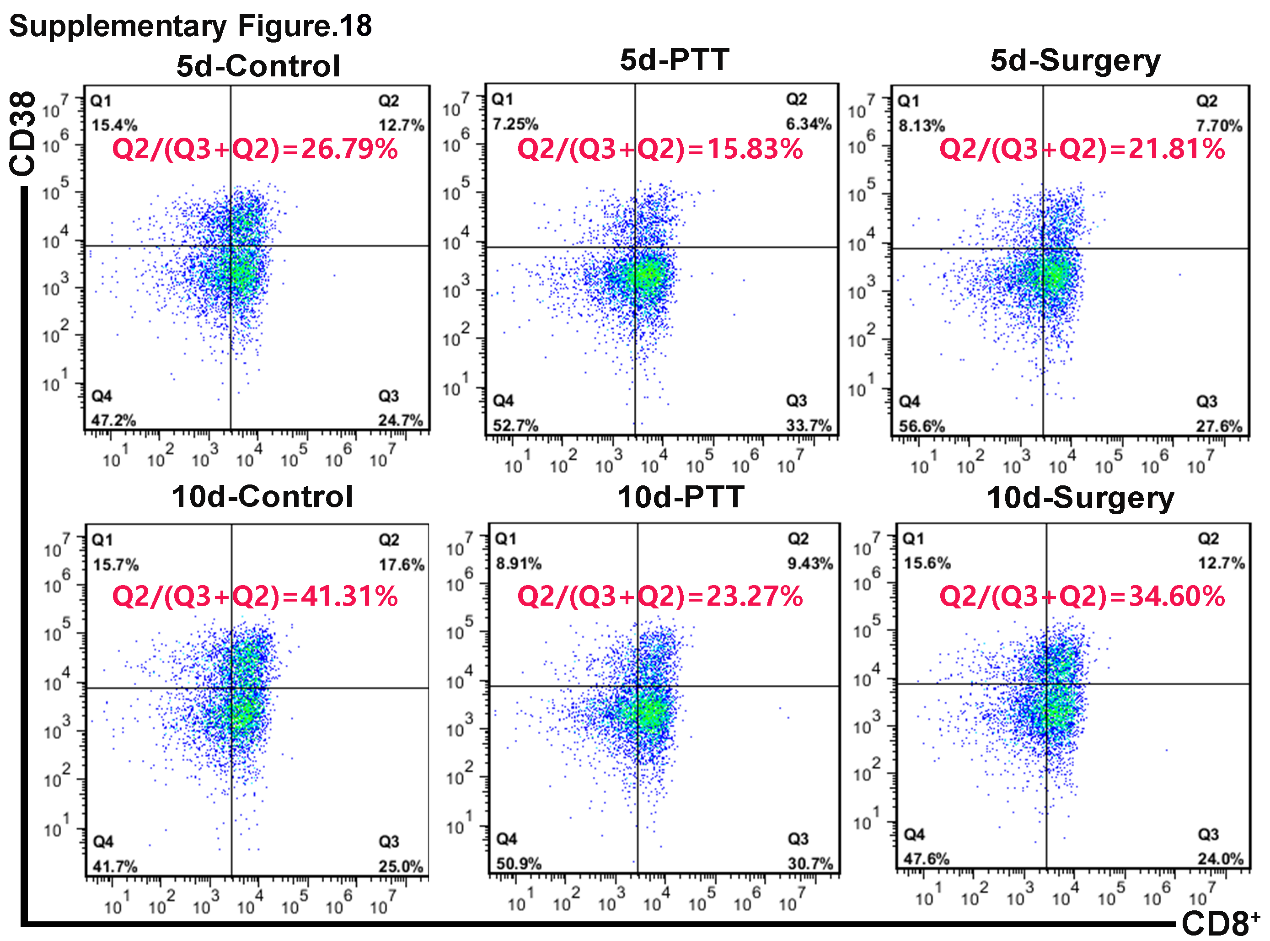


Figure S22. The quantities of CD38 in CD8^+^ T cells in the spleen isolated from the above mice post varied treatment were detected by flow cytometry assay.

**Figure S23**


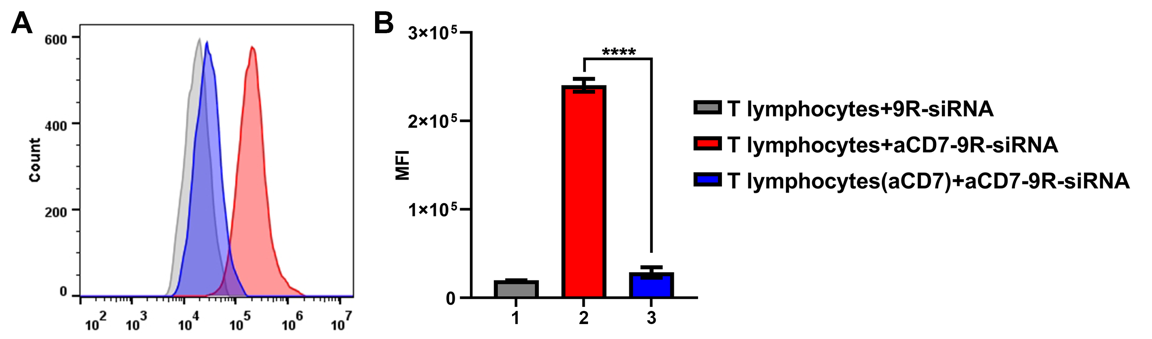


Figure S23. Flow cytometry analysis and the quantities of the uptake of different complex in T lymphocytes. Statistical analysis was conducted by the one-way ANOVA for multiple groups, and the statistical significance was set as ****P < 0.001, n = 5.

**Figure S24**


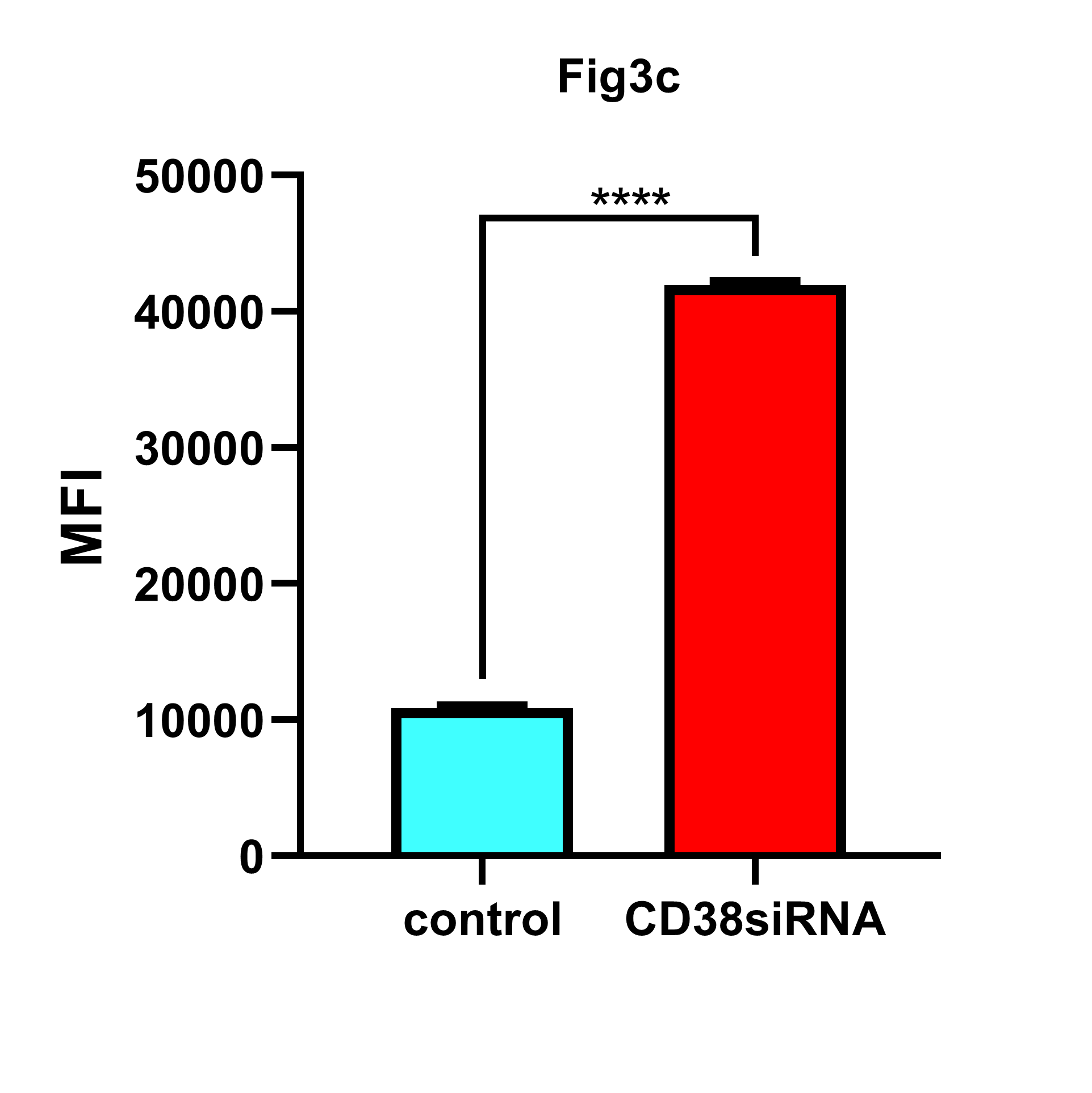


Figure S24. The quantification of T cells transfected with CD38siRNAs (50nM) modified by anti-CD7-9R complex for Figure 3c. Statistical analysis was conducted by the student t test, and the statistical significance was set as *P < 0.05; **P < 0.01; ***P < 0.005; ****P < 0.001, n = 5.

**Figure S25**


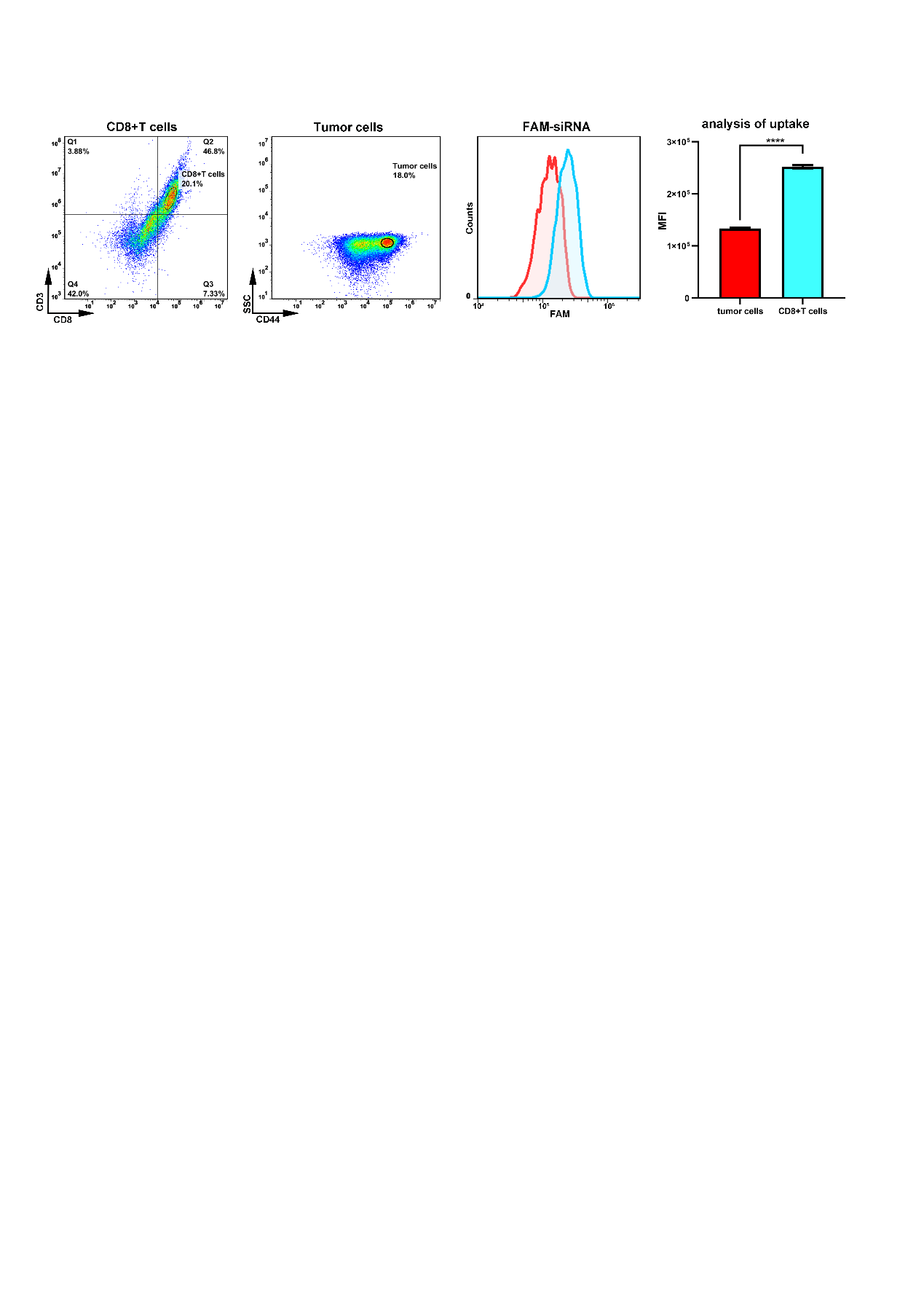


Figure S25. The uptake of different cells in tumor micro environment. CD8+T cells were recognized by CD3 and CD8 double positive, H22 tumor cells were recognized by CD44 positive. Statistical analysis was conducted by the student t test, and the statistical significance was set as ****P < 0.001, n = 5.

**Figure S26**

**
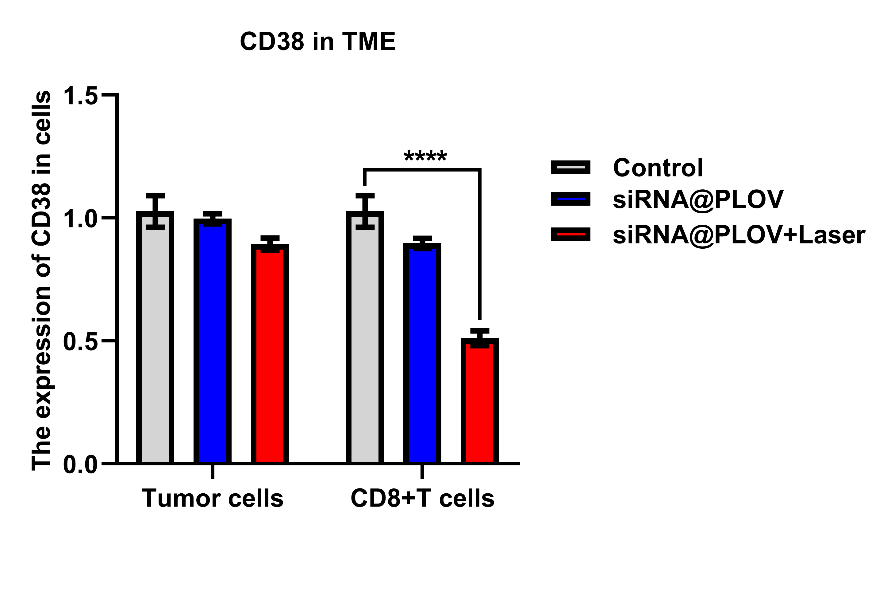
**

Figure S26. The expression of CD38 in CD8+T cells in tumor cells or CD8+T cells after treated with groups. Statistical analysis was conducted by the one-way ANOVA for multiple groups, and the statistical significance was set as *P < 0.05, n = 5.

**Figure S27**


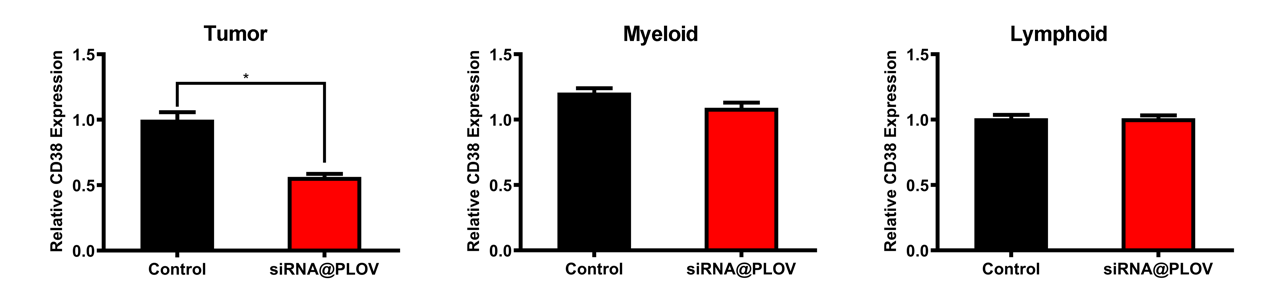


Figure S27. The expression of CD38 in tumor, myeloid or lymphoid after transfected with siRNA@PLOV. Statistical analysis was conducted by the student t test, and the statistical significance was set as *P < 0.05, n = 5.

**Figure S28**


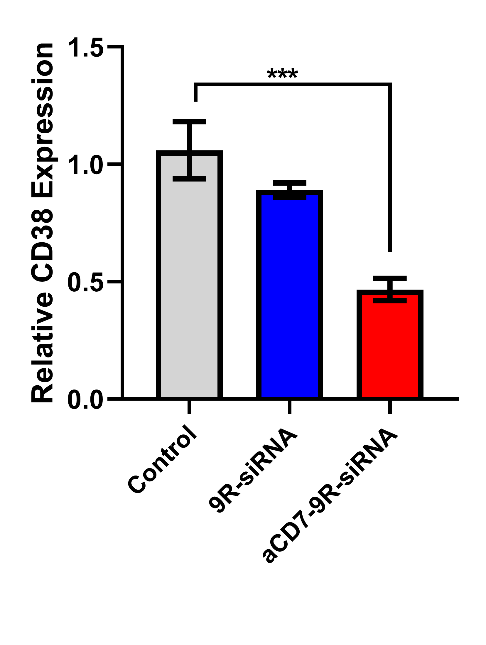


Figure S28. The effect of aCD-7 affect the expression level of CD38 in T cells. Statistical analysis was conducted by the one-way ANOVA for multiple groups, and the statistical significance was set as ***P < 0.005, n = 5.

**Figure S29**


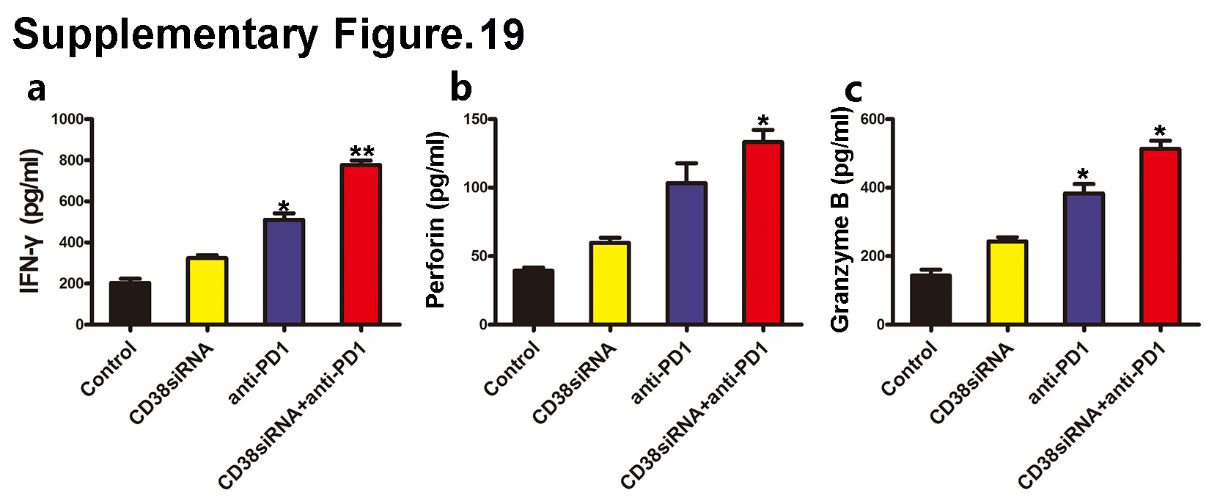


Figure S29. The expression of IFN-γ, perforin and granzyme B were determined by ELISA to evaluate the function of T cells. Statistical analysis was conducted by the one-way ANOVA for multiple groups, and the statistical significance was set as *P < 0.05; **P < 0.01, n = 5.

**Figure S30**


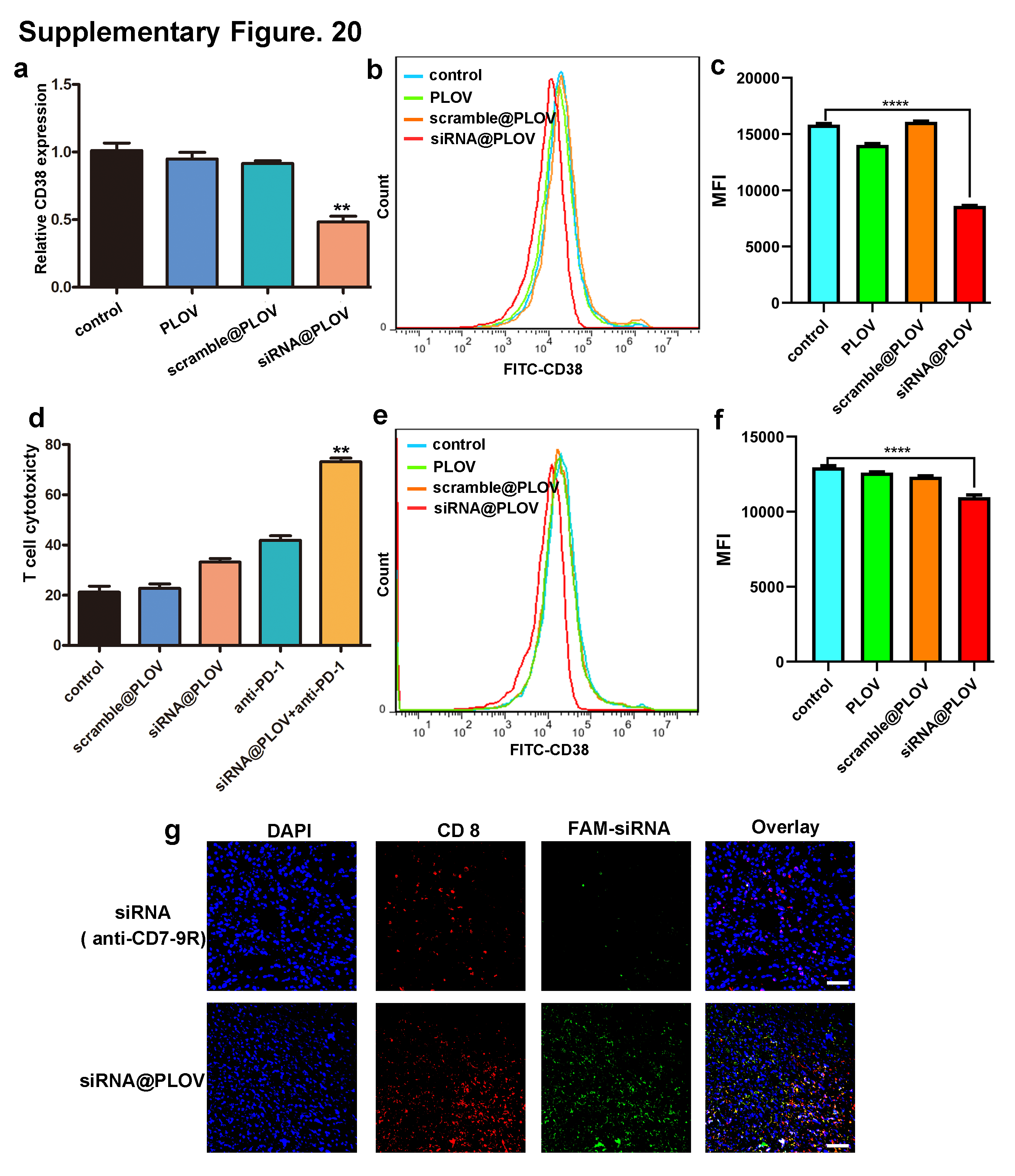


Figure S30. (a) The qRT-PCR analysis of CD38 level in CD8+ T cells transfected with different formulations. (b, c) Flow cytometry analysis and qualification of CD38 expression in CD8+ T cells. (d) The flow cytometry assays of T cells transfected with different formulations. (e, f) Flow cytometry analysis of CD38 expression in CD8+ T cells infiltrated in H22 tumor tissue under different formulations. (g) FAM-siRNA were co-localized with infiltrated CD8+ T cells fluorescence in tumor vasculature. (Red signal, CD8+ T cells; Blue signal, cell nucleus; Green signal, FAM-siRNA). The scale bar is 100 μm. Data are given as mean ± SD (n=5). Statistical analysis was conducted by the one-way ANOVA for multiple groups, and the statistical significance was set as *P < 0.05; **P < 0.01.

**Figure S31**


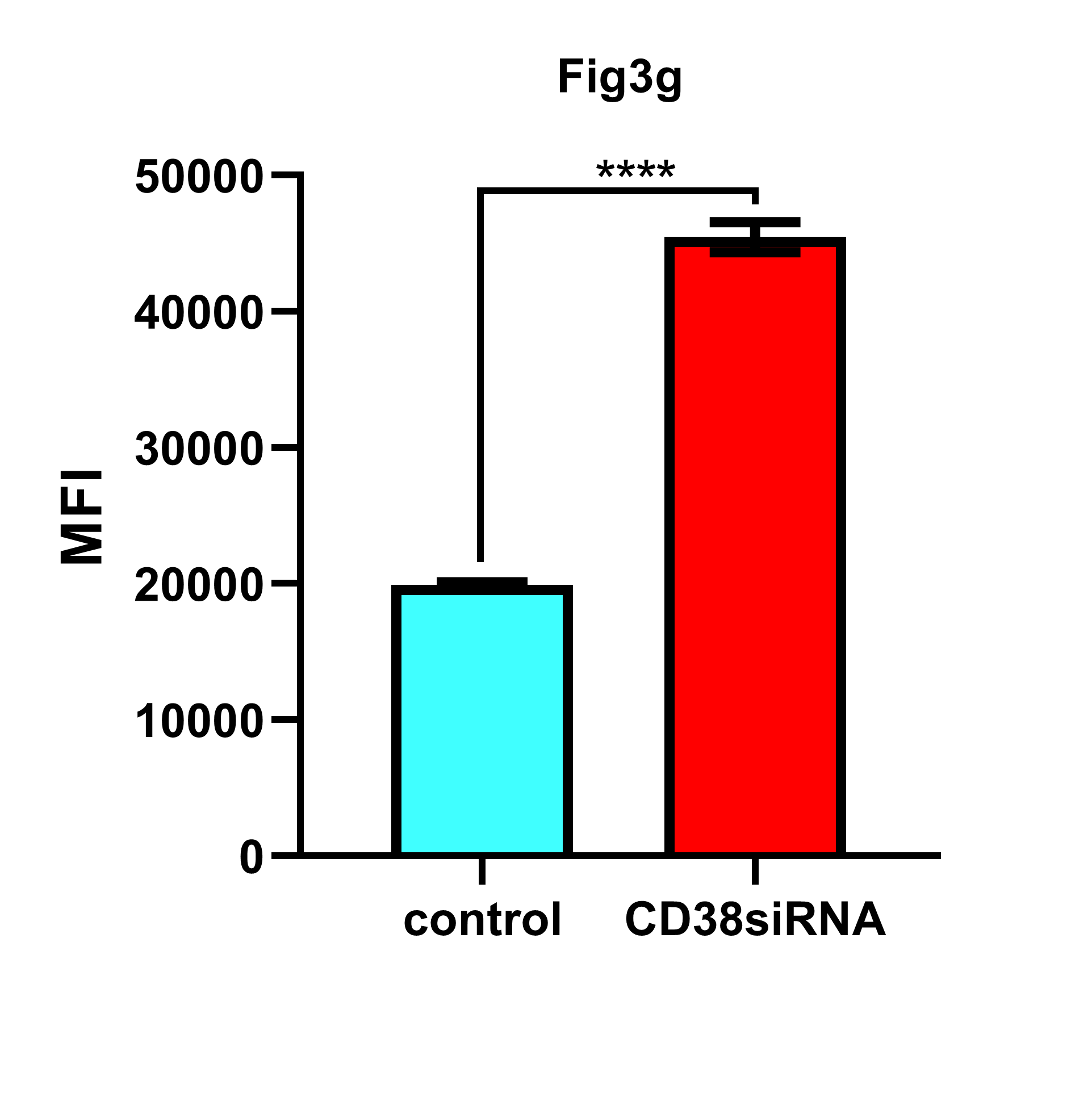


Figure S31. The quantification of CD28 level in CD8+ T cells via CD38 blockade for Figure 3g. Statistical analysis was conducted by the student t test, and the statistical significance was set as *P < 0.05; **P < 0.01; ***P < 0.005; ****P < 0.001, n = 5.

**Figure S32**


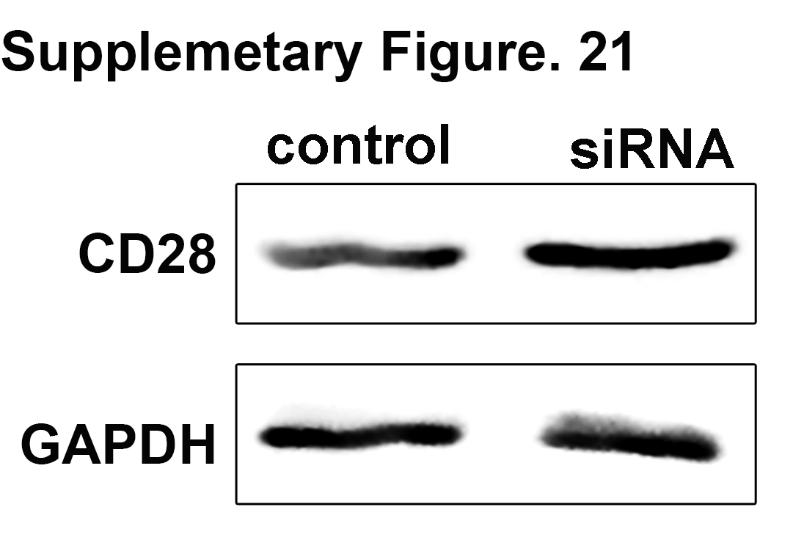


Figure S32. Western blotting assay of CD28 expression post CD38 siRNA incubation.

**Figure S33**


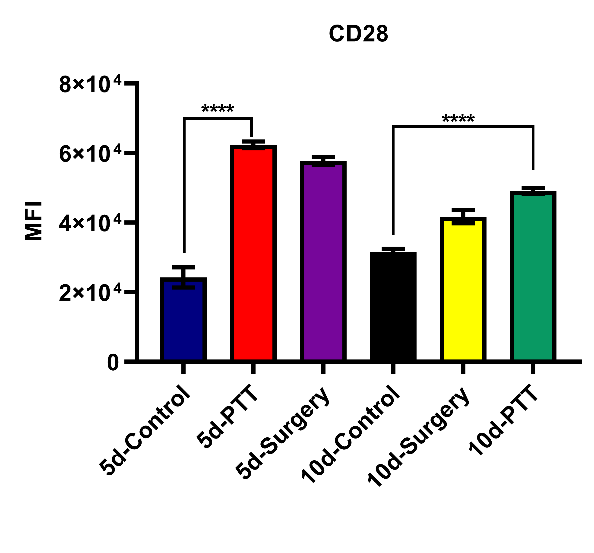


Figure S33. The quantification of The flow cytometry assays of CD28 expression in CD8+ T cells infiltrated in H22 tumor tissue under PTT or surgery treatments 5 or 10 days. for Figure 3h. Statistical analysis was conducted by the one-way ANOVA for multiple groups, and the statistical significance was set as ****P < 0.001, n = 5.

**Figure S34**


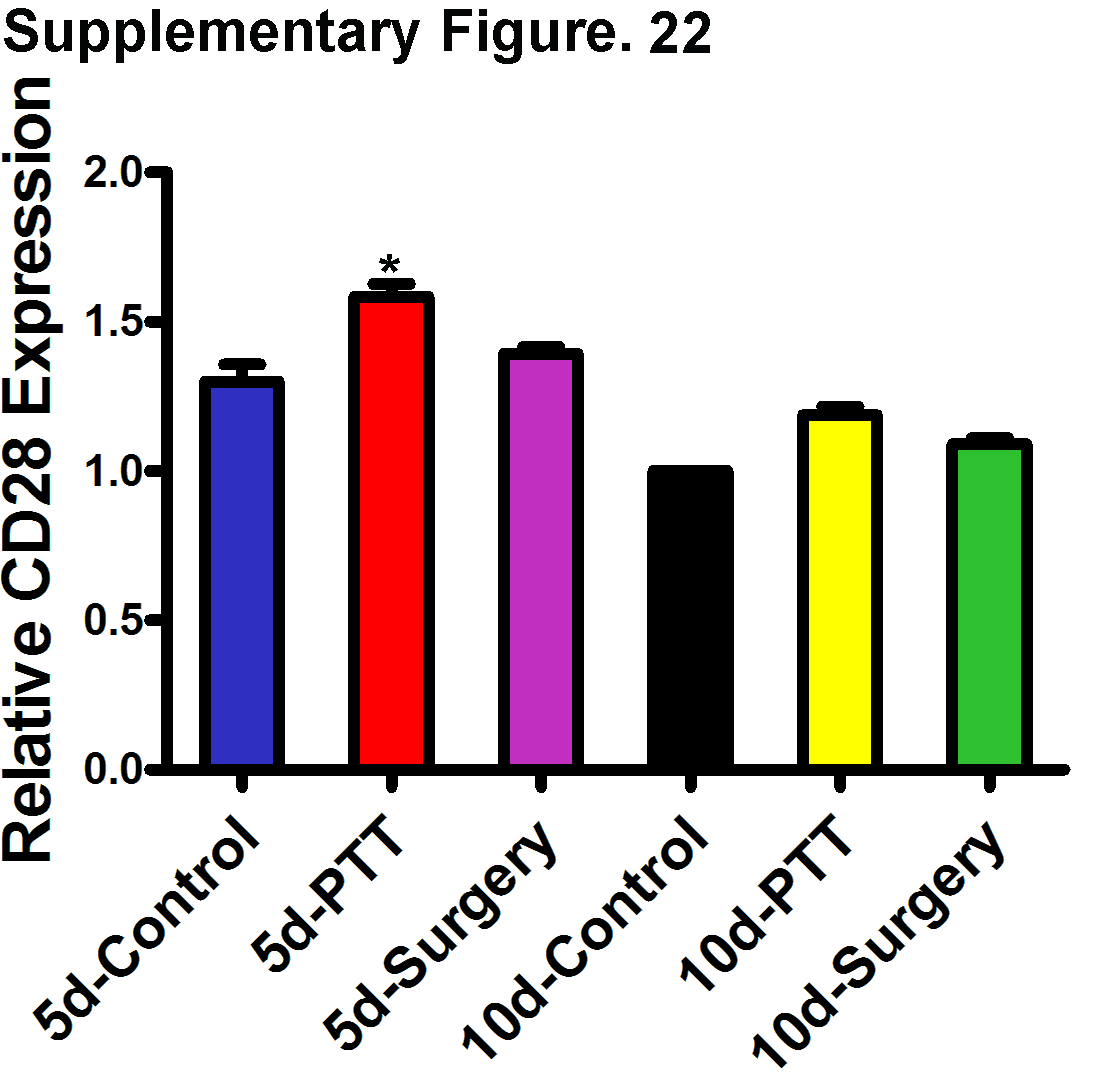


Figure S34. The q-PCR analysis of CD28 expression in the tumor tissues of mice under different treatments. Statistical analysis was conducted by the one-way ANOVA for multiple groups, and the statistical significance was set as *P < 0.05, n = 5.

**Figure S35**


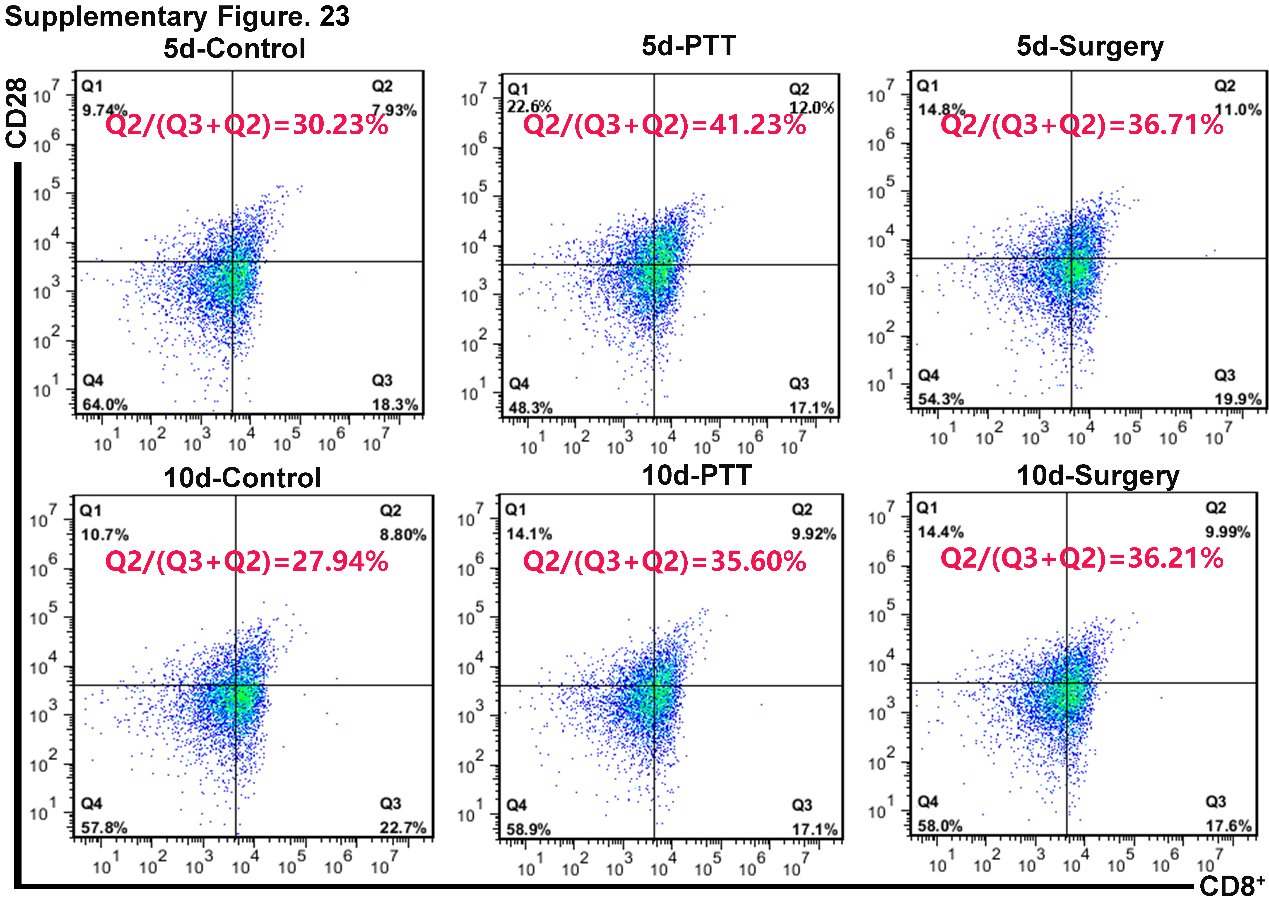


Figure S35. The CD28 level in CD8^+^ T cells infiltrated in the H22 tumor tissues isolated from the mice with described treatments above.

**Figure S36**


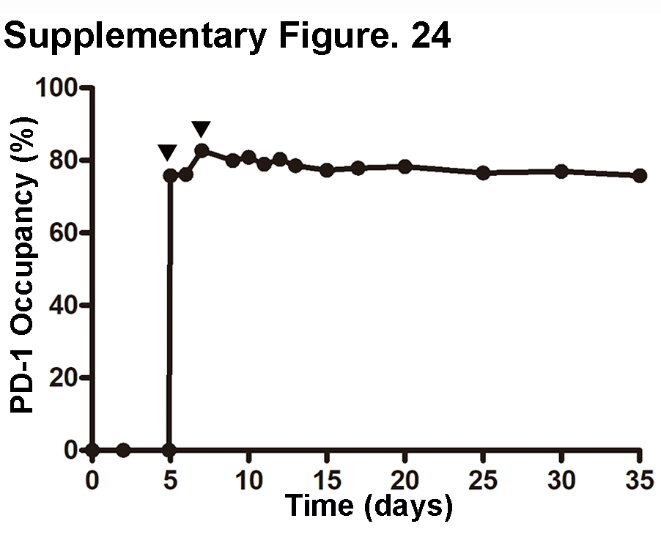


Figure S36. The occupancy of anti-PD-1 with the treatment.

**Figure S37**


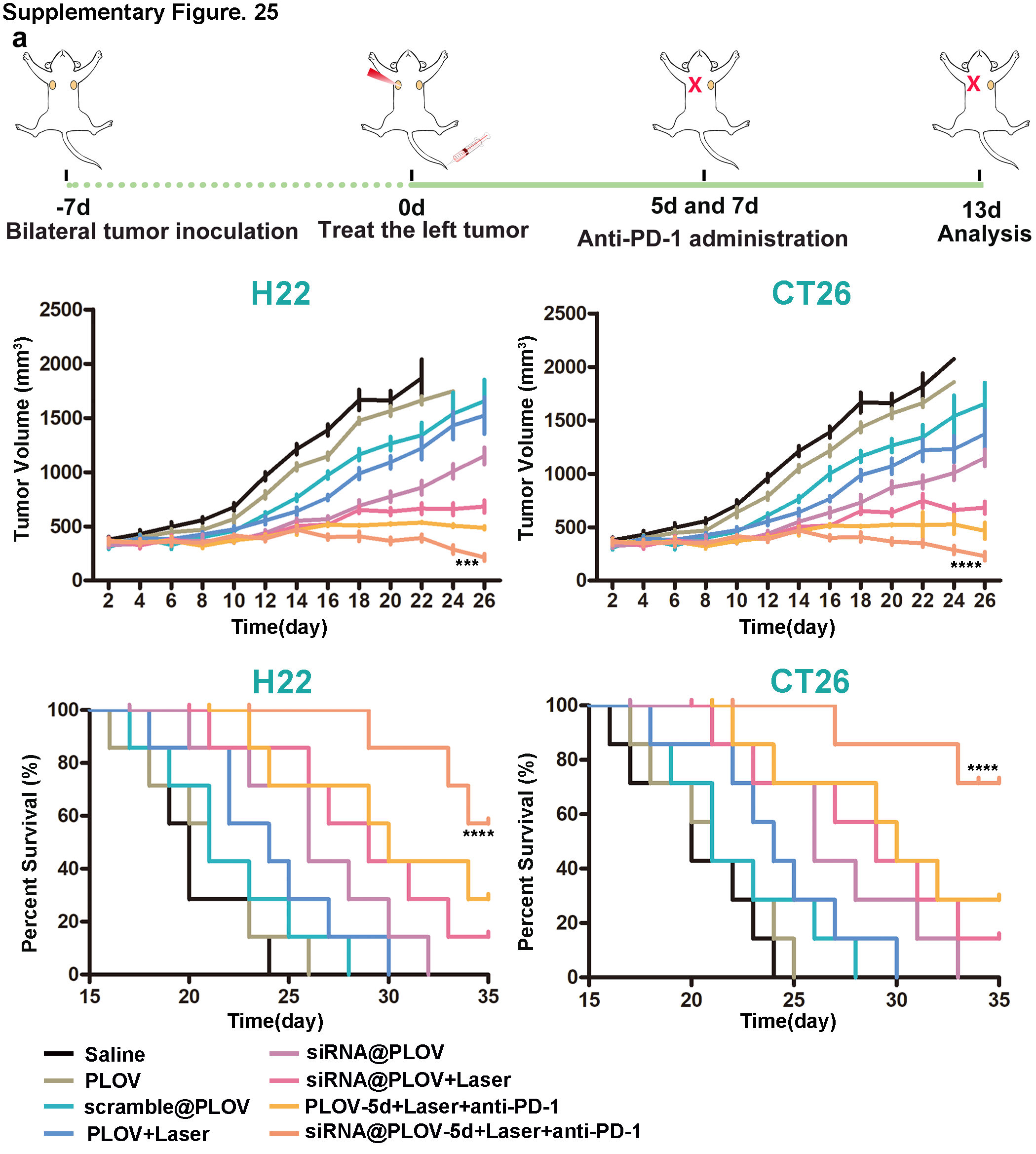


Figure S37. siRNA@PLOV-based PTT combined with anti-PD-1 to suppress distal tumors post elimination of primary tumor (H22 or CT26). (a) Schematic of siRNA@PLOV-based PTT plus anti-PD-1 to suppress tumor growth at distal sites after eradicating primary tumors. (b, d) The distal tumor growth of mice (n=7) with inoculation of secondary H22 (b) or CT26 (d) tumors post corresponding regimens on their primary tumors as described. (c, e) The life-span of mice with inoculation of secondary H22 (c) or CT26 (e) tumors after aforementioned regimens. Statistical analysis was conducted by the one-way ANOVA for multiple groups, and the statistical significance was set as ***P < 0.005; ****P < 0.001, n = 7.

**Figure S38**


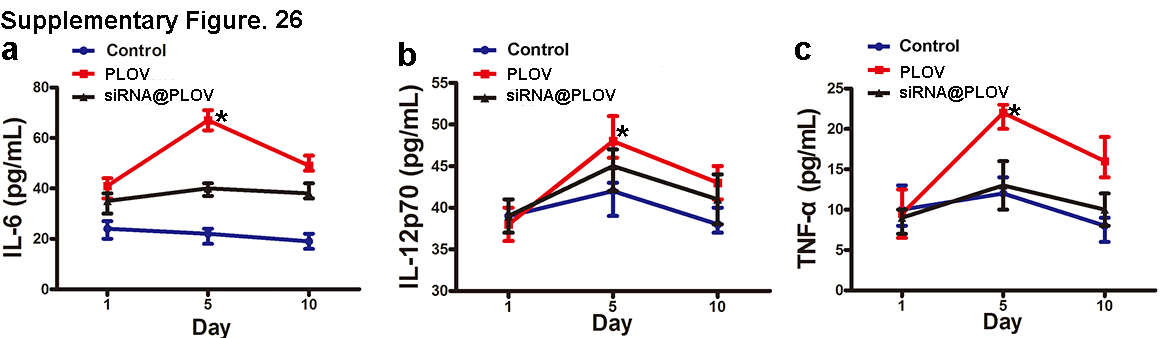


Figure S38. The variation of several cytokines, including IL-6 (a), IL-12p70 (b) and TNF-α (c) in the distal tumor tissues of mice after different approaches measured by ELISA. Statistical analysis was conducted by the one-way ANOVA for multiple groups, and the statistical significance was set as *P < 0.05, n = 7.

**Figure S39**


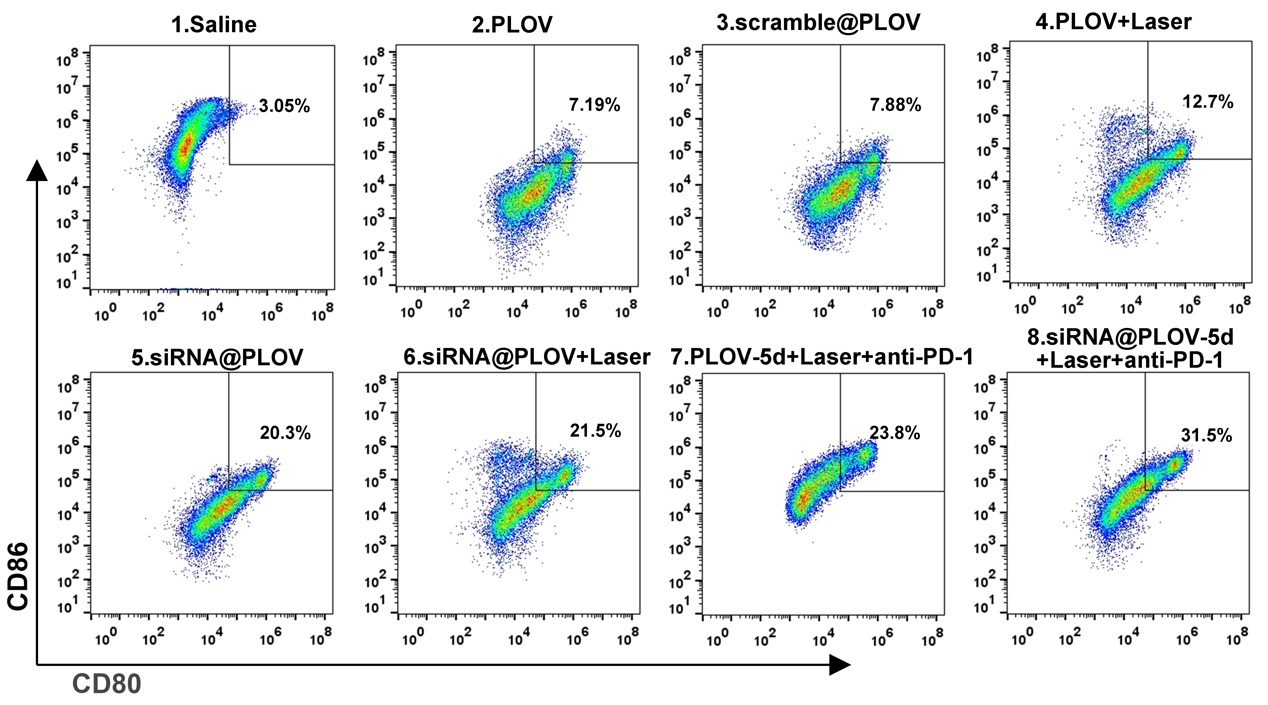


Figure S39. The percentage of **CD80 and CD86 double-positive DC cells** analyzed by flow cytometry in the 2nd H22 tumor tissues in Figure 5. CD80-FITC, and CD86-PE.

**Figure S40**


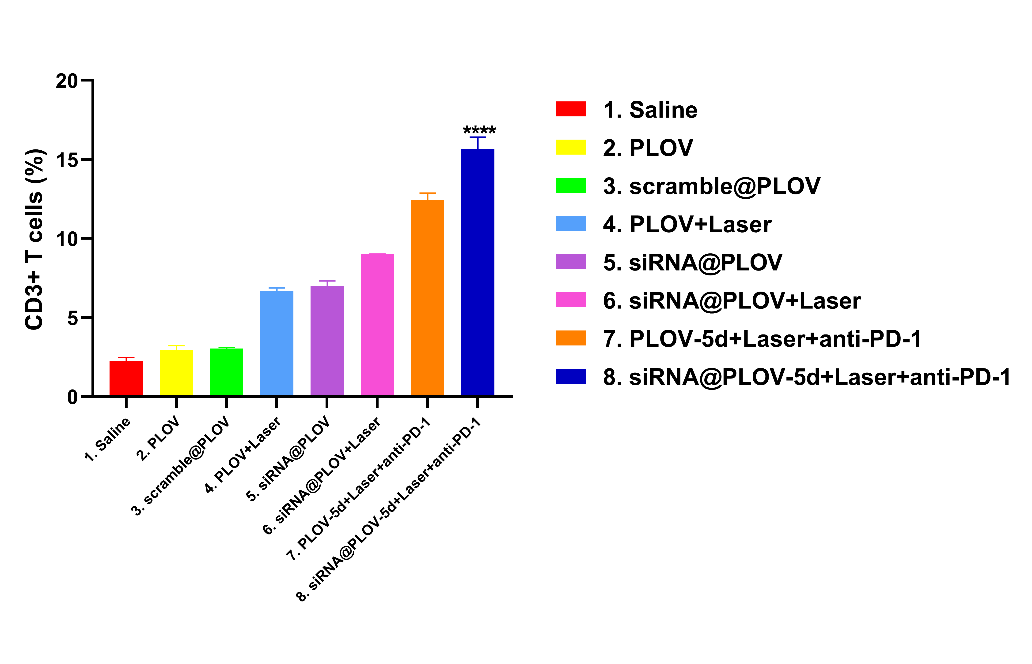


Figure S40. The quantification of **CD3+ T cells** infiltrated in the 2nd H22 tumor tissues under different treatments in Figure 5. Statistical analysis was conducted by the one-way ANOVA for multiple groups, and the statistical significance was set as ****P < 0.001, n = 5.

**Figure S41**


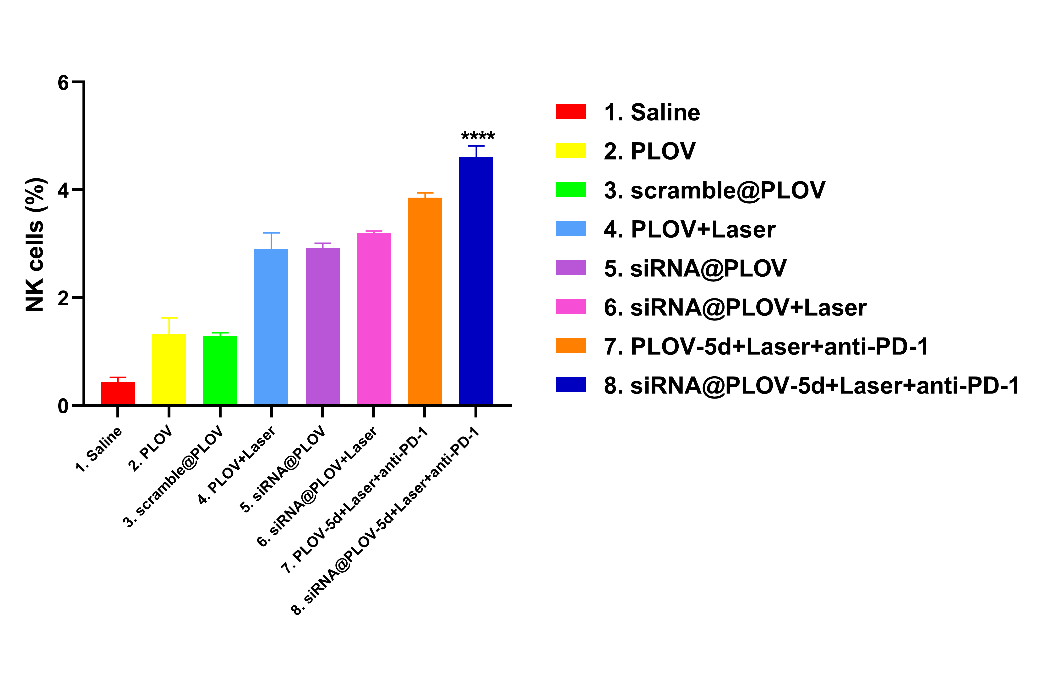


Figure S41. The amounts of **NK cells** infiltrated in the 2nd H22 tumor tissues under different treatments in Figure 5. Statistical analysis was conducted by the one-way ANOVA for multiple groups, and the statistical significance was set as ****P < 0.001, n = 5.

**Figure S42**


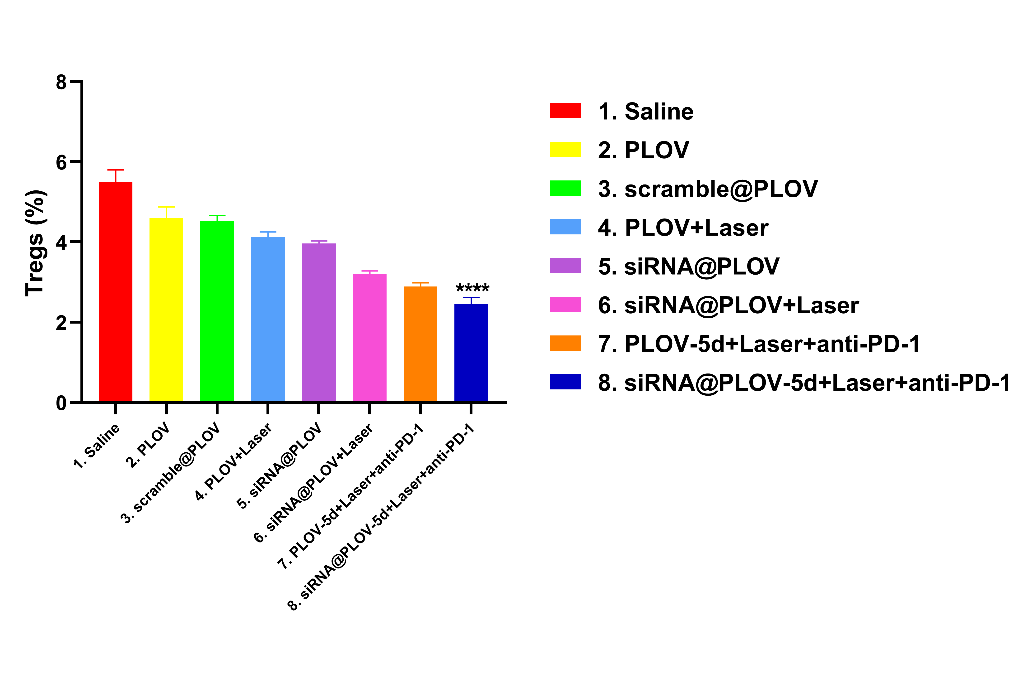


Figure S42. The levels of **Tregs** infiltrated in the 2nd H22 tumor tissues under different treatments in Figure 5. Statistical analysis was conducted by the one-way ANOVA for multiple groups, and the statistical significance was set as ****P < 0.001, n = 5.

**Figure S43**


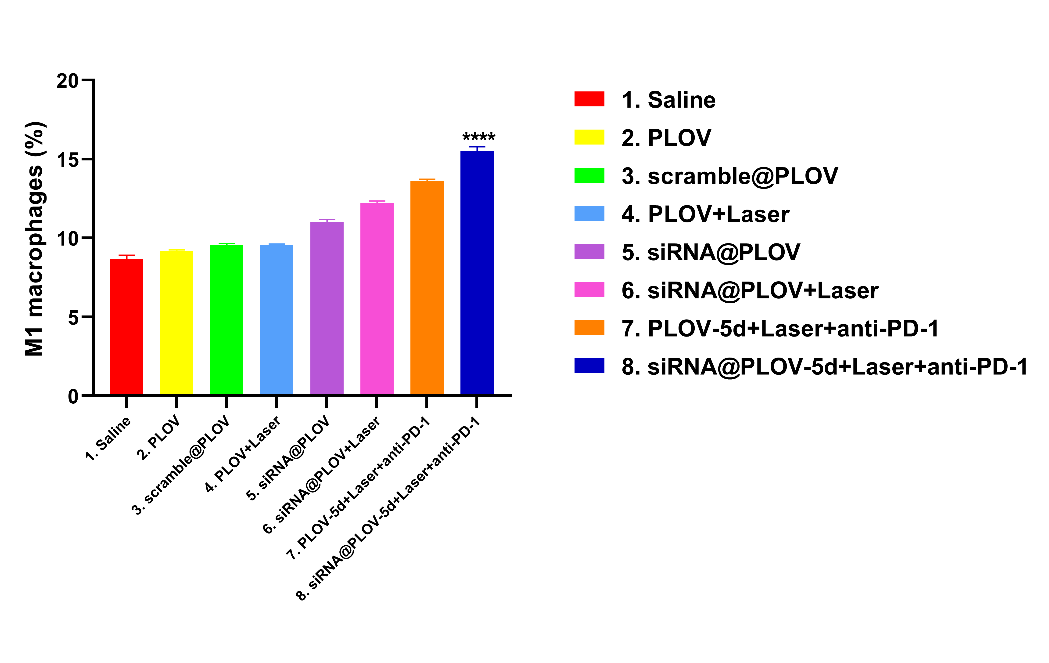


Figure S43. The levels of **M1 Macrophages cells** infiltrated in the 2nd H22 tumor tissues under different treatments in Figure 5. Statistical analysis was conducted by the one-way ANOVA for multiple groups, and the statistical significance was set as ****P < 0.001, n = 5.
